# Ebola virus’ hidden target: virus transmission to and infection of skin

**DOI:** 10.1128/jvi.01300-25

**Published:** 2025-09-12

**Authors:** Paige T. Richards, Anthony M. Fleck, Radhika Patel, Maryam Fakhimi, Dana Bohan, Kathleen Geoghegan-Barek, Anna N. Honko, Allison E. Stolte, Caroline B. Plescia, Caitlin O. Messingham, Samuel J. Connell, Tyler P. Crowe, Francoise A. Gourronc, Ricardo Carrion, Anthony Griffiths, David K. Meyerholz, Aloysius J. Klingelhutz, Robert A. Davey, Kelly N. Messingham, Wendy Maury

**Affiliations:** 1 Department of Microbiology and Immunology, University of Iowa 311821, Iowa City, Iowa, USA; 2 Department of Dermatology, University of Iowa 671906, Iowa City, Iowa, USA; 3 NEIDL, Boston University 1846, Boston, Massachusetts, USA; 4 Texas Biomedical Research Institute 7075, San Antonio, Texas, USA; 5 Department of Pathology, University of Iowa 160416, Iowa City, Iowa, USA; 6 Graduate Program in Immunology, University of Iowa 4083, Iowa City, Iowa, USA; St Jude Children's Research Hospital, Memphis, Tennessee, USA

**Keywords:** filovirus, tropism, skin, animal model, Ebola virus

## Abstract

Ebola virus (EBOV), the causative agent of Ebola virus disease, remains one of the World Health Organization’s top 10 threats to global health. Infectious EBOV virions can be found on the surface of skin late in infection and may be transmitted to others through skin-to-skin contact. We investigate *in vivo* EBOV tropism and the kinetics of virus movement to and from the skin. Increasing viral loads were detected over time in the skin of EBOV-infected non-human primates and mice, with antigen detected in dermal stromal and immune cells. Epidermal cells within and surrounding hair follicles also harbored viral antigen, suggesting a novel mechanism of virus egress to the epidermal surface. During late infection, proinflammatory responses were elevated in infected visceral organs but minimal in the skin despite significant viral loads. We observed similar viral trafficking and cell tropism in the skin of mice intraperitoneally infected with a low containment EBOV model virus, rVSV/EBOV GP, allowing more detailed mechanistic studies. Sites of virus infection in the skin were patchy, with intense focal areas of infection surrounded by uninfected areas. To investigate virus entry into the body through skin, rVSV/EBOV GP was applied to the surface of gently abraded skin to remove the stratum corneum; epidermal keratinocytes were robustly infected with subsequent systemic viral dissemination observed in some mice. Optimal levels of infection within the skin required expression of the phosphatidylserine receptor, AXL. Collectively, our data demonstrate that skin serves as an important organ targeted by EBOV, facilitating virus entry into and egress from the body.

**IMPORTANCE:**

Ebola virus (EBOV) remains one of the World Health Organization’s top 10 threats to global health, despite the availability of a U.S. Food and Drug Administration-approved vaccine. EBOV spreads through human-to-human contact, yet the role of skin in viral transmission remains unclear. Here, we identify skin as a site of EBOV infection, serving as a potential portal for entry into and egress from the body. *In vivo*, infectious virions and viral RNA increased in the skin over time, localizing to dermal myeloid and stromal cells and to cells within and surrounding hair follicles, suggesting a novel mechanism for viral shedding. Skin infection was patchy and associated with minimal inflammation, despite significant viral loads. Using a surrogate EBOV model, we demonstrate that systemic infection can occur following topical administration through abraded skin and requires phosphatidylserine receptor, AXL, for optimal infection of skin. These findings redefine the role of skin in EBOV pathogenesis, with implications for barrier-targeted interventions.

## INTRODUCTION

Sporadic Ebola virus (EBOV) outbreaks occur in Central and Western Africa despite the availability of a highly efficacious U.S. Food and Drug Administration (FDA)-approved vaccine (1). It is therefore not surprising that EBOV remains one of the World Health Organization (WHO)’s top 10 global health threats (2). The largest epidemic, from 2013 to 2015 in West Africa, resulted in approximately 28,000 reported cases and over 11,000 deaths (1). EBOV infection is associated with severe symptoms, including hemorrhagic fever, gastrointestinal distress, multiple organ failure, and cytokine dysregulation that can lead to death (3–6).

EBOV is reported to spread primarily through direct contact with infected bodily fluids, with mucosal transmission considered the main route of infection (3). However, during the West African outbreak, anecdotal evidence suggested that the infectious virus is present on the skin of patients with late-stage infection (7), posing a significant risk of transmission via skin contact. Consistent with this, EBOV RNA and infectious virions have been reported to be evident on the skin of a non-human primate (NHP) after succumbing to infection (8).

Our lab recently developed a transwell human explant model to investigate EBOV infection of skin (9). We demonstrated that EBOV infection via the basal (dermal) surface occurred in a time- and dose-dependent manner, with the virus trafficking through the skin tissue to the apical (epidermal) surface, where infectious virus or viral RNA was often detected. Cells of myeloid, endothelial, epithelial, and stromal origin were infected in dermal tissue, with keratinocytes prominently infected in the epidermis. In this study, we investigate *in vivo* timing and tropism of EBOV infection in the skin of NHPs and mice. We found that infection of skin tissue distal to the site of infection was delayed compared to infection of visceral organs, demonstrating that skin is a late target during EBOV infection. Furthermore, viral loads and proinflammatory responses were more modest in skin than those found in the liver and visceral fat tissue. Within the dermis, stromal, myeloid, and epithelial cells were infected; however, evidence of epidermal keratinocyte infection was limited. If the skin was abraded to remove the stratum corneum, the topically applied virus was able to enter the body at that site and cause severe morbidity. These *in vivo* studies highlight the importance of the skin as a target organ for EBOV, as it allows both entry into and egress from the body.

## RESULTS

### EBOV load increases in NHP skin over the course of infection

NHPs are the gold standard experimental animal for modeling EBOV infection, closely mirroring human disease (10, 11). Prior studies report viral antigen-positive cells in human and NHP skin at late times during infection, and low levels of infectious EBOV persist on macaque skin for up to 3 days postmortem (8, 12). These observations are consistent with the potential for human skin to serve as a conduit for virus transmission, as noted during the West African EBOV epidemic (7, 8, 13). Furthermore, as the skin is the largest organ of the body, an infected area may contribute substantially to systemic infection. While prior reports suggest EBOV traffics to the skin during infection, the kinetics and specific cell types involved *in vivo* remain unclear. To address these knowledge gaps, we analyzed EBOV-infected NHP skin samples obtained from three different studies.

**Fig 1:**
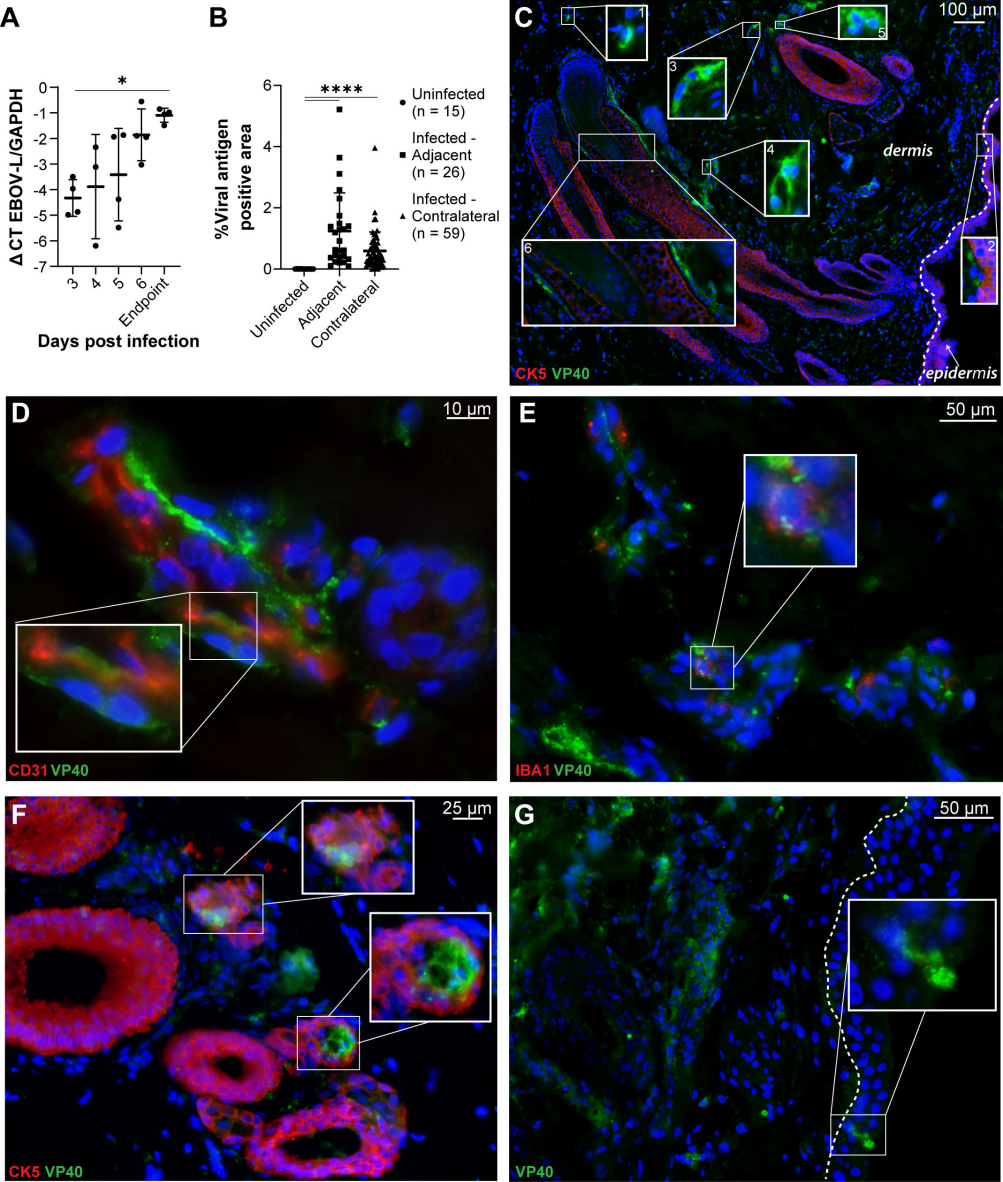
EBOV traffics to and infects NHP skin during systemic infection. (**A**) Viral loads in skin taken from the site of injection in NHPs infected IM with 1,000 PFU of EBOV (Kikwit). Endpoint animals (days 7 and 9) were averaged. Data are expressed on a log_10_ scale as geometric means ± geometric SD. Statistical significance was determined by a nonparametric analysis of variance (ANOVA) test on log-transformed values (**A**, *P < 0.05). (**B–G**) Analysis of immunostained sections from NHPs infected with 100 PFU of EBOV (Kikwit) IM. Tissue sections are from NHPs necropsied at day 6 (**B and C**) or 7 (**B, D–G**) when endpoint criteria were met. (**B**) Percent area of viral antigen positivity (VP40 or EBOV GP): uninfected (*n* = 15), infected skin adjacent to the injection site (*n* = 26), and infected skin from the contralateral regions (*n* = 59), analyzed by ImageJ and statistical significance, was determined by a nonparametric ANOVA test (**B**, ****P < 0.0001). (**C–G**) Immunofluorescence images of EBOV-infected NHP skin showing EBOV VP40 (green), cell markers as noted (red), and 4’,6-diamidino-2-phenylindole, blue (DAPI). Representative images from two independent experiments (*n* = 5 NHPs per experiment). (**C–F**) Skin from the contralateral arm. (**G**) Skin adjacent to the site of infection. (**C**) Robust VP40 antigen was detected in cells in the deep dermis and cells adjacent to CK5^+^ keratinocytes along hair follicles and the epidermis (magnified in insets). (**D**) Colocalization of VP40^+^ with CD31^+^ dermal endothelial cells. (**E**) VP40 detected in IBA1^+^ dermal macrophages, as were IBA1^+^/ VP40^−^ and IBA1^−^/VP40^+^ cells. (**F**) VP40 colocalized with CK5^+^ cells as well as adjacent CK5^−^ cells in hair follicles within the dermis. (**G**) VP40^+^ cells (magnified in the inset) were present in the epidermis. The dotted line in panels **B and F** denotes the location of the epidermal-dermal junction. Scale bar (μm) is indicated in each panel. Brightness and contrast were adjusted uniformly across the entire image for visualization purposes. Routine staining controls are shown in Fig. S1D and E, H and I. Single channels and colocalization analyses for each image are shown in Fig. S2.

In one set of NHP skin samples, viral loads were evaluated in skin samples from EBOV-infected NHPs euthanized over a 9-day infection. Macaques infected intramuscularly (IM) with 1,000 PFU of EBOV (Kikwit) were necropsied on 3, 4, 5, and 6 days post-infection (dpi) or at endpoint (day 7 or day 9). Typical mean time to death in EBOV-infected macaques is between 6 and 9 days (14). Details of clinical findings and virus loads in other tissues from these animals were described previously (10). Viral RNA levels in skin at the injection site increased over time (Fig. 1A), with levels of EBOV L gene detection reaching statistical significance when animals were near endpoint and providing evidence of ongoing replication in the skin. These findings are consistent with those of the earlier study with these EBOV-infected NHP, which reported increasing viral replication in most tissues over time (10). Notably, by both our evaluation of normalized EBOV L RNA levels to host GAPDH values and Alfson et al. genome equivalents determinations (10), virus in the skin at the injection site was evident by day 3 of infection but was not detected in serum until day 4. This suggests that skin supports local virus replication before systemic dissemination.

While formalin-fixed, paraffin-embedded (FFPE) NHP skin tissues from this study were not available, FFPE NHP skin was obtained from two independent NHP EBOV (Kikwit) studies. In one instance, male and female rhesus macaques were inoculated IM with 100 PFU of EBOV (Kikwit) and sacrificed on day 6 or 7 (Fig. 1). In a second study, FFPE skin was obtained from two rhesus macaques that served as infected controls in an EBOV (Kikwit) antiviral study. Animals were inoculated IM with 1,000 PFU and reached endpoint on days 6 and 8 (Fig. S1). We sectioned and analyzed all available FFPE tissues from the skin near the site of infection (adjacent), as well as skin taken from the contralateral arm, to assess the distribution of EBOV antigen (VP40 or glycoprotein [GP]). Co-staining of cell markers included CK5 (keratinocytes/epithelial cells), CD140ab (fibroblasts/smooth muscle/pericytes), CD31 (endothelial cells), IBA1 (macrophages/microglia), and CD11b (myeloid cells/macrophages).

Across sections, the quantity of viral antigen positivity varied from zero to more than 5% of the total area of the skin section (Fig. 1B). Numerous viral antigen-positive cells were evident by day 6 (Fig. 1C). Insets in Fig. 1C highlight widespread clusters of EBOV VP40^+^ cells present deep within the dermis (Fig. 1C, inset 1) to the epidermal-dermal junction (Fig. 1C, inset 2). EBOV antigen-positive cell morphology included both elongated (Fig. 1C, insets 3 and 4) and rounded cells (Fig. 1C, inset 5). This suggests a diversity of cell types supporting EBOV infection within the skin. EBOV antigen was frequently found in cells adjacent to CK5^+^ hair follicles (Fig. 1C, inset 6), indicating that mesenchymal cells involved in follicle maintenance were infected (15).

Since NHPs exhibit hallmarks of Ebola virus disease, such as vascular leakage (10, 14), we examined viral colocalization within the dermal vasculature of the skin. EBOV antigen was present in CD31^+^ cells, as well as in cells adjacent to the blood vessel (Fig. 1D, inset), suggesting that dermal endothelial cells and cells surrounding the endothelium support infection in NHPs. Viral antigen also colocalized with cells staining for the macrophage markers IBA1 (Fig. 1E, inset) and CD11b (Fig. S1A) within the dermis. This is consistent with myeloid cells being early and sustained EBOV targets (16–18). VP40 was also observed in CK5^+^ cells surrounding hair follicles and in structures morphologically consistent with sebaceous glands (Fig. 1F; Fig. S1B and C). Infection of the epidermis was observed infrequently (Fig. 1G). The limited EBOV infection of the epidermal layer was unexpected, since our earlier study demonstrated prominent epidermal infection at late stages of infection of human skin explants (9).

Shown in Fig. S2 are single-channel images of the immunofluorescent images shown in Fig. 1C through G. Figure S3 displays the single-channel images of the supplementary images in Fig. S1. A summary of the number of sections analyzed for the frequency of viral antigen-positive and specific cell marker positive sections per animal is summarized in Table 1. For all staining experiments, the specificity of immunostaining was demonstrated using infected tissue sections stained with secondary antibody only and the absence of EBOV antigen staining in uninfected tissues (Fig. S1D and E, respectively).

**TABLE 1:** Summary of positive cell marker and viral antigen staining in all NHP skin sections used in this study^*a*^

NHP no.	Skin site relative to inoculum	Cell marker antibody	No. of cell marker positive/total sections	No. of virus positive/total sections
Female 1	Adjacent	IBA1	3/3	3/3
	Contralateral	IBA1	6/7	7/7
	Adjacent	CK5	2/2	2/2
	Contralateral	CK5	10/10	10/10
	Adjacent	PDGFR-β	5/5	5/5
	Contralateral	CD45	n.d./1	1/1
	Contralateral	CD11b	3/4	4/4
Male 1	Adjacent	IBA1	2/2	2/2
	Contralateral	IBA1	9/9	9/9
	Adjacent	CK5	10/10	10/10
	Contralateral	CK5	9/9	9/9
	Adjacent	CD31	3/4	4/4
	Contralateral	CD31	2/2	2/2
	Contralateral	PDGFR-β	1/1	1/1
Female 2	Contralateral	CD31	7/7	7/7
	Contralateral	CD45	n.d./3	3/3
	Contralateral	CD11b	4/4	4/4
Male 2	Contralateral	IBA1	2/2	0/2
Column totals			78/85	83/85

^*a*^ n.d. indicates not detected.

To further assess NHP epidermal susceptibility to EBOV-GP-mediated infection, we used an explant model with 1 cm^2^ rhesus skin sections maintained at the air/liquid interface on semipermeable transwells, similar to our prior studies with human skin explants (9). A biosafety level 2 (BSL-2) EBOV model virus, recombinant vesicular stomatitis virus that encodes EBOV GP and green fluorescent protein (GFP) in place of the native G glycoprotein (rVSV/EBOV GP), was inoculated into the basal media in the presence of a type I IFN inhibitor, B18R. We have established in human skin explants that rVSV/EBOV GP tropism in the presence of an interferon inhibitor closely mimics that of BSL-4 EBOV (9). After removal of the input virus, basal media were collected on the days noted to monitor viral titers, and a subset of explants was harvested on day 8 for immunostaining. Viral titers within the basal media increased over time (Fig. S1F), and viral antigen was detected in both the dermis and epidermis, as observed in the *in vivo* infections (Fig. S1G). Uninfected and secondary control NHP skin explant sections confirmed specific staining (Fig. S1H and I). Notably, viral antigen was observed within the epidermis in these infections, co-localizing with CK5^+^ cells, indicating the infection of NHP keratinocytes. Together, these findings identify diverse cell populations within NHP skin supporting EBOV infection.

### ma-EBOV traffics to the mouse skin during infection

To investigate *in vivo* EBOV infection of the skin of another animal model, we infected and monitored mice over a 5-day infection with mouse-adapted EBOV (ma-EBOV). The mouse infections enabled a larger sample size and more precise kinetic analysis. While mice do not fully recapitulate human filovirus disease, as hemorrhagic manifestations or intravascular coagulation are not evident during ma-EBOV, this experimental model provides valuable insights into virus tropism and the kinetics of infection (11, 19, 20). Six-week-old female C57Bl/6 mice were infected intraperitoneally (IP) with 1,000 focus-forming units (FFU) of ma-EBOV, which typically results in lethality between days 6 and 8 (14, 19). Importantly, ma-EBOV is only lethal in immunocompetent mice when delivered IP (14, 20). Mice (*n* = 10/day) were euthanized on days 0–5 of infection, approximately 2–3 days before the typical mean time to endpoint in this model (14, 19).

**Fig 2:**
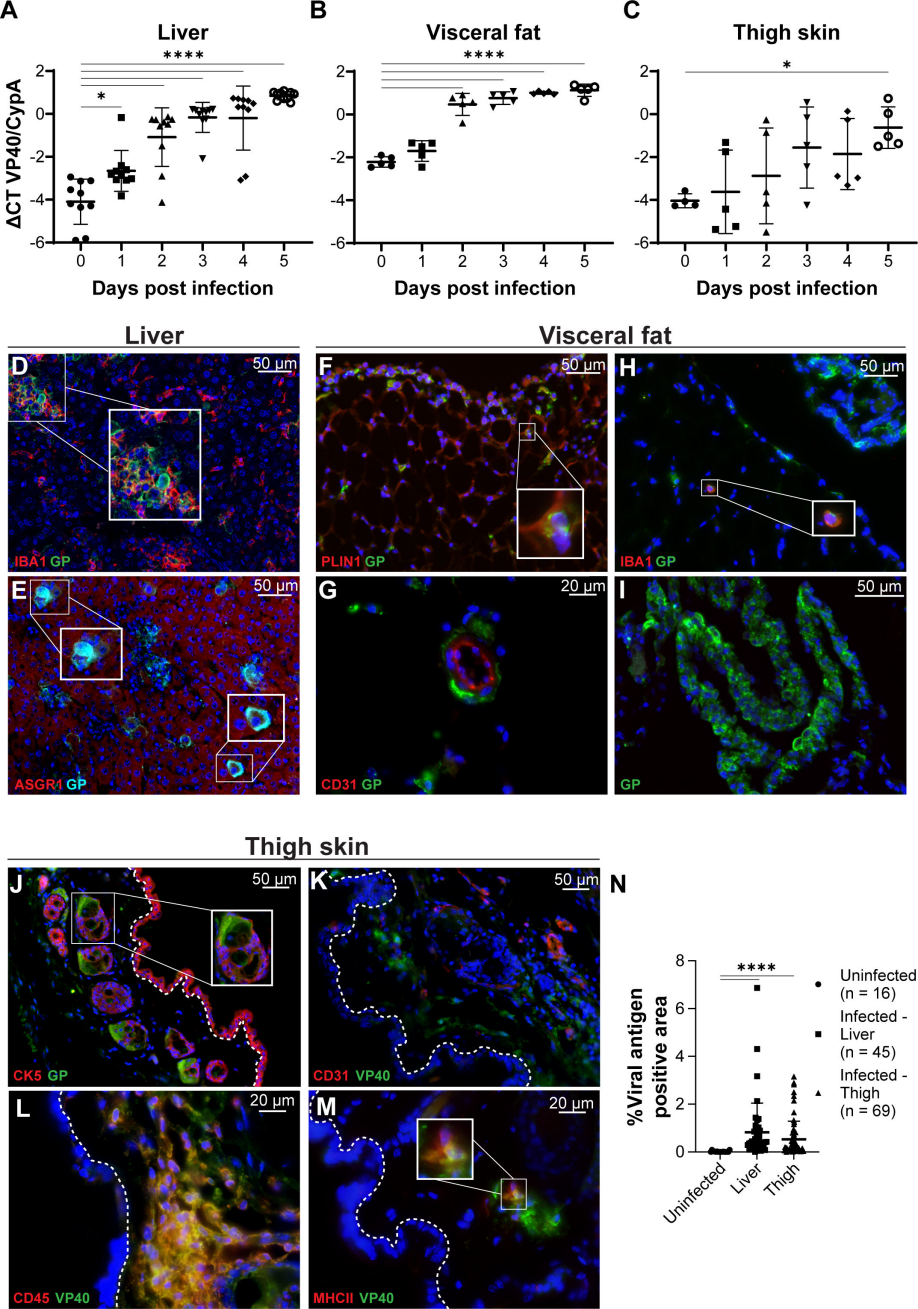
ma-EBOV kinetics and viral cell tropism in mice. Female C57BL/6 mice were infected IP with 1,000 FFU of ma-EBOV. EBOV expression was evaluated on days 0–5 post-infection (*n* = 4–10/day/tissue). (**A–C**) Viral loads in liver (**A**), visceral fat (**B**), and thigh skin (**C**) were determined by real-time quantitative reverse transcription PCR (RT-qPCR) for EBOV VP40, normalized to the housekeeping gene, mouse cyclophilin A (CypA). Data are expressed on a log_10_ scale as geometric mean ± geometric SD. A one-way analysis of variance (ANOVA) determined statistical significance (**A–C**, *P < 0.05; ****P < 0.0001). (D–M) Viral antigen (VP40 or EBOV GP, green) and cell-specific marker (indicated in red) with DAPI (blue) immunostaining of liver (**D and E**), visceral fat (**F–I**), and thigh skin (**J–M**) from mice at 5 dpi. The dotted line in panels **J–M** indicates the epidermal-dermal junction. Scale bar (μm) is shown in each panel. Brightness and contrast were adjusted uniformly across the entire image for visualization purposes. Single channels and colocalization analyses for each of the **J–M** panels are shown in Fig. S5. (**N**) Percent area of viral antigen positivity (VP40 or EBOV GP) in uninfected (*n* = 16), day 5 liver (*n* = 45), and day 5 thigh skin (*n* = 69) sections, quantified using ImageJ. A nonparametric ANOVA test determined statistical significance (**N**, ****P < 0.0001).

The liver, a known target of EBOV, served as a positive control. Viral RNA levels increased significantly in a time-dependent manner, reaching statistical significance by day 1 of infection (Fig. 2A). Similarly, visceral fat near the peritoneal injection site showed increasing viral load over time (Fig. 2B). Thigh skin, distal to the injection site, showed highly variable viral load between days 1 and 4, but levels were consistently elevated by day 5 (Fig. 2C). Other tissues that were assessed for viral load included skin at the site of infection (belly skin), subcutaneous fat lining the belly skin, and skin taken from the back (Fig. S4A through C). Both belly skin and subcutaneous fat exhibited significant increases in viral load over time. In contrast, virus levels in back skin were more variable, and the average virus load was not significantly enhanced during the 5-day study. This variability may reflect delayed trafficking to distal dorsal skin regions.

To identify infected cell types, we performed immunostaining on FFPE tissue sections from day 4 or 5 of infection. Cell-specific markers included ASGR1 (hepatocytes), CK5 (keratinocytes/epithelial cells), PLIN1 (adipocytes), CD31 (endothelial cells), CD45 (immune cells), MHC II (antigen-presenting cells), CD3 (T cells), and IBA1 (macrophages/microglia). Secondary control and uninfected tissue sections confirmed specific staining (Fig. S4D through F, respectively).

We detected EBOV antigen in liver macrophages (Kupffer cells) and hepatocytes at day 5 post-infection (Fig. 2D and E). Focal areas of infected macrophages were observed (Fig. 2D, inset), aligning with prior ma-EBOV studies (18), but infected hepatocytes appeared more uniformly distributed in the tissue. In visceral fat adjacent to the peritoneal injection site, viral antigen colocalized with PLIN1^+^ cells, providing direct evidence of adipocyte infection *in vivo* (Fig. 2F). While CD31^+^ endothelial cells in the visceral fat did not colocalize with viral antigen, infected cells were observed adjacent to those cells surrounding blood vessels (Fig. 2G). This aligns with previous findings that endothelial cell infection is rare outside the liver and lymphoid tissues in ma-EBOV-infected mice (21). Although macrophages are known EBOV targets, co-staining of IBA1 and EBOV was only occasionally detected in visceral fat (Fig. 2H).

The most prominent viral antigen staining was observed in cells within the peritoneal ligaments, which are folds of serous membranes connecting visceral organs (Fig. 2I; Fig. S4F, white arrow; Fig. S4G and H, red arrows). Hematoxylin and eosin (H&E)-stained sections of visceral ligaments had notable areas of mononuclear inflammation (Fig. S4G and H, red arrows), as did parts of the lining of the visceral peritoneum (Fig. S4G and H, black arrows). Rare sites of the inflammation were also associated with fat necrosis (Fig. S4I, black arrows).

In distal skin sites, such as the thigh, abundant clusters of viral antigen^+^ cells were observed near the epidermal-dermal interface at day 5 (Fig. 2J through M), similar to NHP skin (Fig. 1). EBOV antigen colocalized within cells adjacent to hair follicles, possible sebaceous glands, and cells distributed throughout the dermis (Fig. 2J; Fig. S4J). Similar to the visceral fat, viral antigen did not colocalize with endothelial cells in the thigh skin (Fig. 2K). By day 4, clusters of infected cells appeared in the dermis adjacent to CD45^+^ cells, with an increase in frequency and intensity by day 5 (Fig. S4K; Fig. 2L, respectively). Only a subset of EBOV-infected cells within these dermal clusters expressed MHC II (Fig. 2M). Since MHC II is upregulated in activated macrophages but is also expressed by other antigen-presenting cells (22, 23), the lack of co-staining indicates that many infected cells were either not macrophages or macrophages that did not upregulate MHC II in this setting. Shown in Fig. S5 are the single-channel images corresponding to images shown in Fig. 2J through M and Fig. S4J and K, demonstrating colocalization in some cells and an absence of colocalization in others.

The quantification of viral antigen staining revealed variable levels of virus antigen (~0%–7% of the total area of the skin section) in both the thigh skin and liver sections, with marginally higher levels in the liver (Fig. 2N). Table 2 summarizes the quantification of specific cell markers and viral staining in thigh skin from multiple sections of ma-EBOV-infected mice.

**TABLE 2:** Summary of positive cell marker and viral antigen staining in all ma-EBOV mouse thigh skin sections used in this study^*a*^

Mouse no.	Days post infection	Cell marker antibody	No. of cell marker positive/total sections	No. of virus positive/total sections
Mouse 1	3	CD45	1/1	1/1
Mouse 1	4	CD45	3/4	3/4
Mouse 2	4	CD45	2/2	2/2
Mouse 3	4	CD45	3/3	2/3
Mouse 1	5	CD45	6/6	6/6
		MHCII	4/4	2/2
		CK5/CD45	n.d./3	3/3
		IBA1	n.d./7	7/7
Mouse 2	5	CD45	1/1	1/1
		F4/80	1/1	1/1
Mouse 3	5	CD31	5/5	5/5
		CK5	4/4	4/4
Mouse 4	5	CD45	8/8	8/8
		MHCII	8/8	8/8
		CD31	7/7	7/7
		CK5	6/6	5/6
		CD3	n.d./2	1/2
		CD11b	n.d./4	4/4
		IBA1	n.d./3	3/3
Column totals			59/79	73/79

^*a*^ n.d. indicates not detected.

As a second mouse model of EBOV infection, *interferon alpha-beta receptor* knockout (KO; *Ifnar ^–/–^*) mice were infected IP with 1,000 FFU WT EBOV (Mayinga) and euthanized when endpoint criteria were met. To evaluate the viral antigen distribution in this skin at late times of EBOV infection, FFPE samples of the back skin were completely sectioned through, and sections spaced every 30 µm (0–390 μm) were immunostained. There were clusters of viral antigen^+^ cells in some sections, which often localized to the superficial dermis and structures consistent with hair follicles (Fig. S6A, 30 μm section). Other sections showed decidedly less viral antigen^+^ staining (Fig. S6A, 210 μm). The quantification revealed that the percent area of viral antigen-positive intensity varied throughout the tissue (Fig. S6B), reflecting a non-uniform distribution of infection.

### The proinflammatory transcription profiles associated with ma-EBOV infection in visceral organs are not evident in infected distal skin sites

While proinflammatory environments can help control EBOV infection, immunomodulatory environments—often associated with M2-like macrophage polarization, tissue repair, immune regulation, and dampened inflammatory response—can enhance viral replication and infection (24, 25). Prior studies demonstrated that EBOV upregulates proinflammatory cytokines in visceral host tissues despite encoding proteins that inhibit host antiviral responses (3, 21, 26–35). However, our observation that infected immune cells in the skin did not notably upregulate MHC class II expression suggested that EBOV infection may not elicit inflammation equally across all tissues.

**Fig 3:**
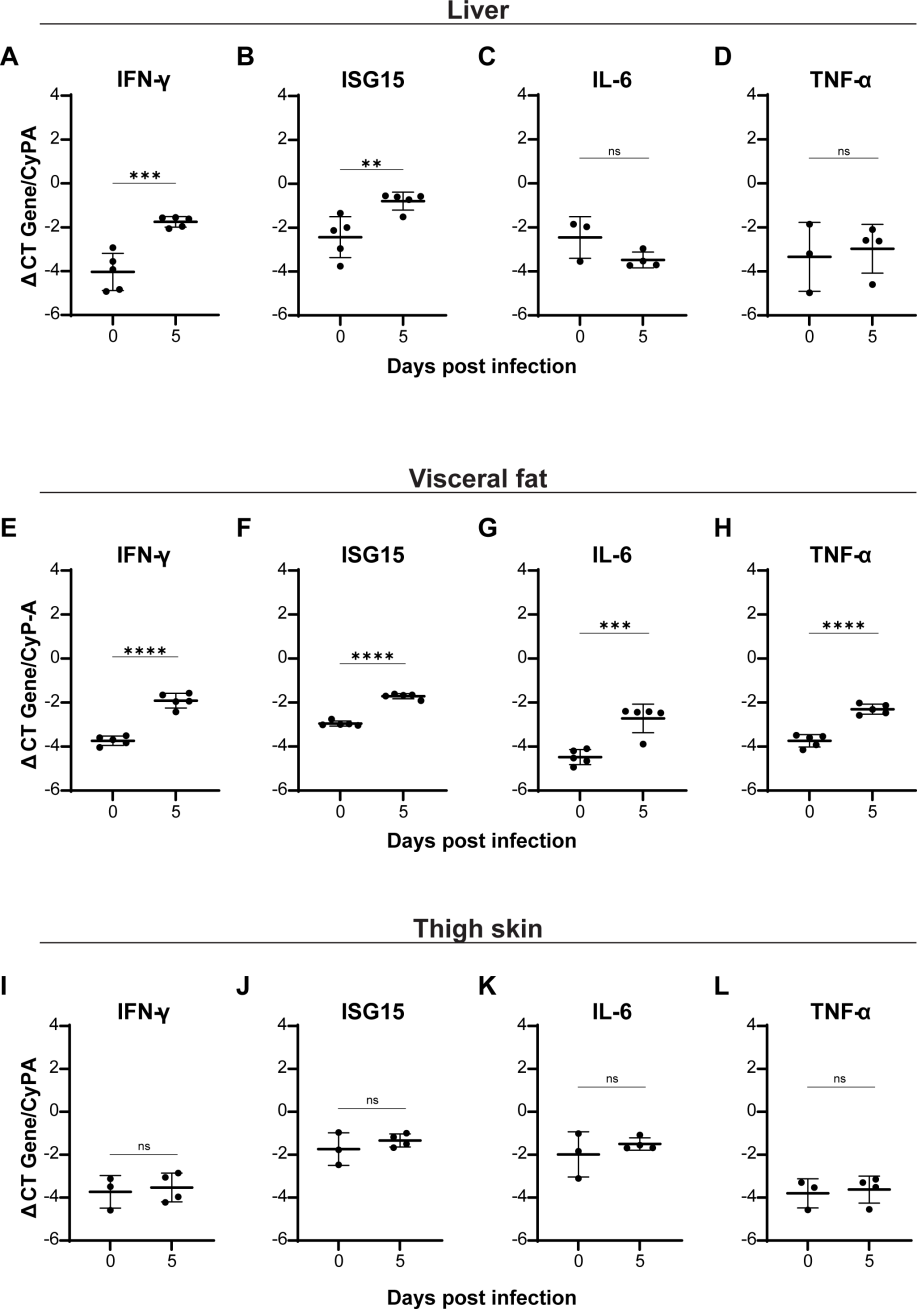
Inflammatory profiles of ma-EBOV-infected mouse tissues. C57BL/6 mice were infected as described in Fig. 2. The expression of proinflammatory gene transcripts (IFN-γ, ISG15, IL-6, and TNF-α) was evaluated by RT-qPCR, and transcript expression was normalized to the housekeeping gene, CypA. Transcript levels were measured in the liver (**A–D**), visceral fat (**E–H**), and thigh skin (**I–L**) at 0 and 5 dpi. Data are shown on a log_10_ scale as geometric means ± geometric SD. Student’s *t*-test determined statistical significance (**P < 0.01; ***P < 0.001; ****P < 0.0001).

To assess inflammatory profiles of different ma-EBOV infected mouse tissues, we measured transcript expression of several proinflammatory markers on day 5 of infection, including interferon gamma (IFN-γ), interferon stimulated gene product 15 (ISG15), interleukin 6 (IL-6), and tumor necrosis factor alpha (TNF-α), as well as immunomodulatory markers such as inflammatory zone 1 (Fizz1) and arginase 1 (Arg1; Fig. 3; Fig. S7).

In the liver, proinflammatory gene transcripts, ISG15 and IFN-γ, were elevated (Fig. 3A and B), whereas IL-6 and TNF-α remained unchanged (Fig. 3C and D). Immunomodulatory gene transcripts, Fizz1 and Arg1, were also unchanged (Fig. S7A and B). In the visceral fat, proinflammatory gene transcripts were significantly increased, which is consistent with our report of adipocyte upregulation of proinflammatory cytokine RNAs following EBOV and rVSV/EBOV GP infection (Fig. 3E through H) (22). Interestingly, Fizz1 expression decreased at day 5 of infection, while Arg1 increased (Fig. S7C and D). Since Arg1 can be upregulated in response to inflammation as a counter-regulatory mechanism, elevated Arg1 may be in response to the proinflammatory tissue environment (36).

Thigh skin did not show significant changes in any inflammatory or immunomodulatory transcripts (Fig. 3I through L; Fig. S7E and F), despite the high viral load and robust antigen staining. Similarly, distal back skin had no significant changes in any inflammatory or immunomodulatory transcripts (Fig. S7G through L). Subcutaneous fat associated with the belly skin, by contrast, showed strong upregulation of several proinflammatory genes, except IL-6, and no changes in immunomodulatory genes (Fig. S7M through R). Belly skin also had elevated expression of IFN-γ and ISG15 (Fig. S7S through V), while Fizz1 and Arg1 displayed mixed regulation (Fig. S7W and X). Together, these data highlight robust proinflammatory responses in visceral tissues, accompanied by a striking absence of such responses at distal skin sites, despite the presence of viral RNA and antigen.

### rVSV/EBOV GP infection models EBOV skin infection in mice

**Fig 4:**
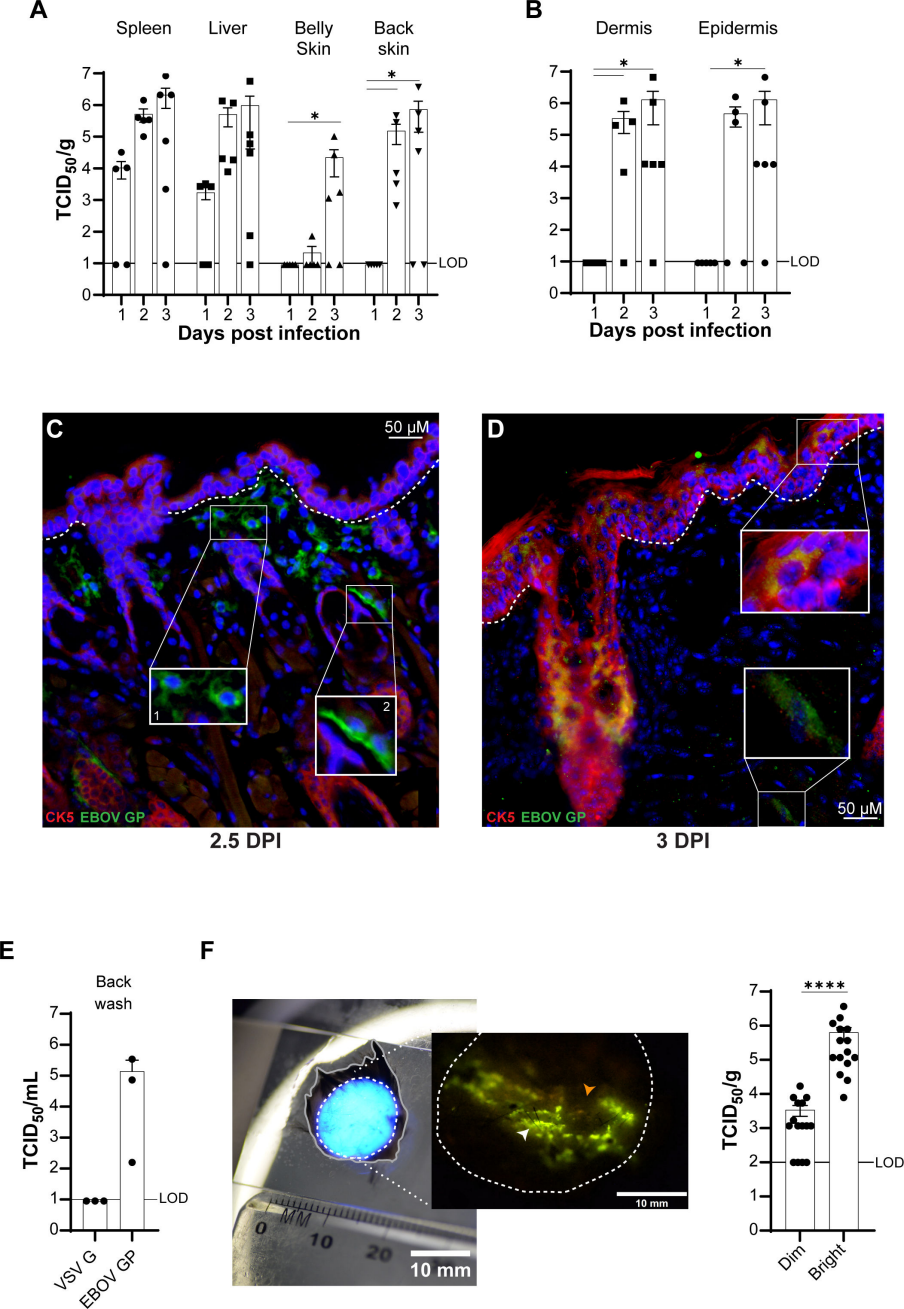
rVSV/EBOV GP targets similar cells as ma-EBOV in distal skin. C57BL/6 *Ifnar^–/–^* mice were infected IP with 500 TCID_50_ of rVSV/EBOV GP (*n* = 3–6). (**A and B**) Viral titers were measured from 1 to 3 dpi in spleen, liver, belly skin, and back skin (**A**), and in enzymatically separated epidermal and dermal layers of back skin (**B**). (**C and D**) Sections of cheek skin were immunostained at 2.5 and 3 dpi for EBOV GP (green), CK5 (red), and DAPI (blue). The dotted line denotes the epidermal-dermal junction. Infected CK5^−^ rounded cells (**C**, inset 1), elongated dermal cells near follicles (**C**, inset 2), CK5^+^ epidermal cells (**D**, top inset), and CK5^−^ dermal cells (**D**, bottom inset) were observed. Brightness and contrast were adjusted uniformly across the entire image for visualization purposes. Scale bar (μm) is shown in each panel. (**E**) Infectious virus on the apical epidermal surface of mouse back skin was sampled by applying 100 µL of Dulbecco’s phosphate-buffered saline (DPBS) to the shaved area of mice 3 dpi with either rVSV/EBOV GP or rVSV/G and measured on Vero E6 cells. (**F**) Representative fluorescence image of shaved back skin (inset) showing GFP-bright (white arrow) and GFP-dim (orange arrow) regions. Punch biopsies from these regions were titered. Data are expressed on a log_10_ scale as mean ± SEM. Statistical significance was determined by a one-way analysis of variance (ANOVA) (**A and B**, *P < 0.05) and Student’s *t* test (**F**, ****P < 0.0001).

To investigate EBOV-GP-specific tropism in more detail, we assessed rVSV/EBOV GP infection in C57BL/6 *Ifnar^–/–^* mice. rVSV/EBOV GP is lethal to these mice within 3–5 dpi when delivered IP, progressing more rapidly than EBOV (24, 37, 38). Viral titers were detectable in the spleen and liver within 1 day, reaching high levels by days 2–3 (Fig. 4A). Surprisingly, virus replication was limited in skin at the injection site (belly skin), but high titers were observed in distal back skin by days 2–3. To evaluate the relative contributions of the epidermis and dermis during viral dissemination, we enzymatically separated these layers from the back skin of mice. The separated epidermal and dermal layers had detectable viral loads in most samples at day 2 that increased further by day 3 post-infection (Fig. 4B). In contrast, mice infected with a lethal dose (LD) of rVSV/G did not have appreciable viral titers in belly or back skin by day 3, despite having similar titers in the spleen as rVSV/EBOV-GP-infected mice (Fig. S8A and B). This suggests that the observed timing of virus trafficking to the skin is driven by EBOV-GP-mediated mechanisms.

To evaluate the tropism, skin cells targeted by rVSV/EBOV GP were assessed by immunofluorescence. Secondary control and uninfected tissue sections confirming specific staining, as well as single-channel images with colocalization analysis for Fig. 4 are shown in Fig. S8C through F. Immunostaining at 2.5 days post-infection revealed EBOV GP antigen-positive cells distributed in the dermis and adjacent to hair follicles (Fig. 4C), suggesting that rVSV/EBOV GP effectively models ma-EBOV tropism but spreads more rapidly to the skin. By day 3, viral antigen was also robustly expressed in CK5^+^ epithelial cells in the epidermis and lining hair follicles as well as in dermal cells (Fig. 4D). The strong expression of viral proteins in CK5^+^ epidermal keratinocytes suggests that rVSV/EBOV GP may have enhanced tropism for the epidermis.

Prior studies in human skin explants suggested that the infectious virus traffics through skin and can be found on the skin’s surface (9). To evaluate that in an *in vivo* setting, the mouse back skin was shaved to remove the fur 5 days prior to IP injection. Dulbecco’s phosphate-buffered saline (DPBS; 100 µL) was placed for ~2 minutes on the shaved back of mice 3 days after IP infection with either rVSV/G or rVSV/EBOV GP. Following the brief incubation, the DPBS was collected from the epidermis and infectious virus titered (Fig. 4E). The virus was present on the skin surface of mice infected with rVSV/EBOV GP, but not rVSV/G, demonstrating rVSV/EBOV GP traffics to the surface of the epithelium at 3 dpi when mice are inoculated IP (Fig. 4E). Consistent with our findings in ma-EBOV-infected mouse skin (Fig. 2M; Fig. S5F), we observed that dermal MHC II expression was modest and only partially colocalized with viral antigen (Fig. S8G and H).

Variability in virus loads and titers in skin from mice infected with EBOV or rVSV/EBOV GP suggested that viral infection was not uniformly distributed, even at late times of infection. Instead, some areas sampled had high virus load, whereas others had little or none (Fig. 2C; Fig. S4A and C; Fig. 4A and B). Hence, we postulated that foci of virus infection occur in the skin. To investigate this, *Ifnar^−/−^* mice were infected IP, verified for systemic infection on 3 dpi (Fig. S8I), and a large piece of back skin was harvested from mice. A fluorescence laser was used to excite the viral-encoded GFP and visualize locations within the skin positive for virus infection. Fluorescent “hot spots” of virus infection were evident (Fig. 4F, inset), with intense areas of GFP (Fig. 4F, inset, white arrow) and areas with dim or no apparent GFP (Fig. 4F, inset, orange arrow). Small biopsies were taken from GFP-dim or GFP-bright areas. Virus titers were significantly higher in the GFP-bright areas than in the GFP-dim areas (Fig. 4F, right panel). These data confirm that rVSV/EBOV GP infection of the skin is patchy, with infectious hot spots and uninfected areas, and explain the wide variation in detection of virus in the skin.

To further characterize these focal infections, we examined whether infected cells were more abundant in the epidermis or dermis. Back skin was harvested from rVSV/EBOV GP-infected mice at 3 dpi, and GFP-bright regions were isolated. Dermal and epidermal layers were separated, and cells within those layers were dissociated. GFP^+^ cells were identified by flow cytometry. Representative flow plots show gating strategies used to identify the epidermal (bottom) and dermal (top) populations (Fig. S8J). Quantification revealed that approximately 21.7% of cells in the epidermis were GFP-positive, compared to the 7.7% of dermal cells (Fig. S8K). These findings support rVSV/EBOV GP infection of keratinocyte-rich regions of the skin. Since the epidermis is a fraction of the size and weight of its corresponding dermis, a greater percentage of infected epidermal cells present late times of rVSV/EBOV GP infection could explain the equivalent titers observed in epidermal and dermal tissues shown in Fig. 4B.

### rVSV/EBOV GP infection of abraded skin can cause systemic infection

Anecdotal reports suggest that person-to-person transmission of EBOV via skin contact prompted us to examine whether topical infection of the skin can mediate systemic infection (7, 13, 39). To determine if skin could serve as an initial target of EBOV-GP-mediated infection (outside-in trafficking), NHP skin explants and *Ifnar^–/–^* mice were infected with our low-containment virus, rVSV/EBOV GP.

**Fig 5:**
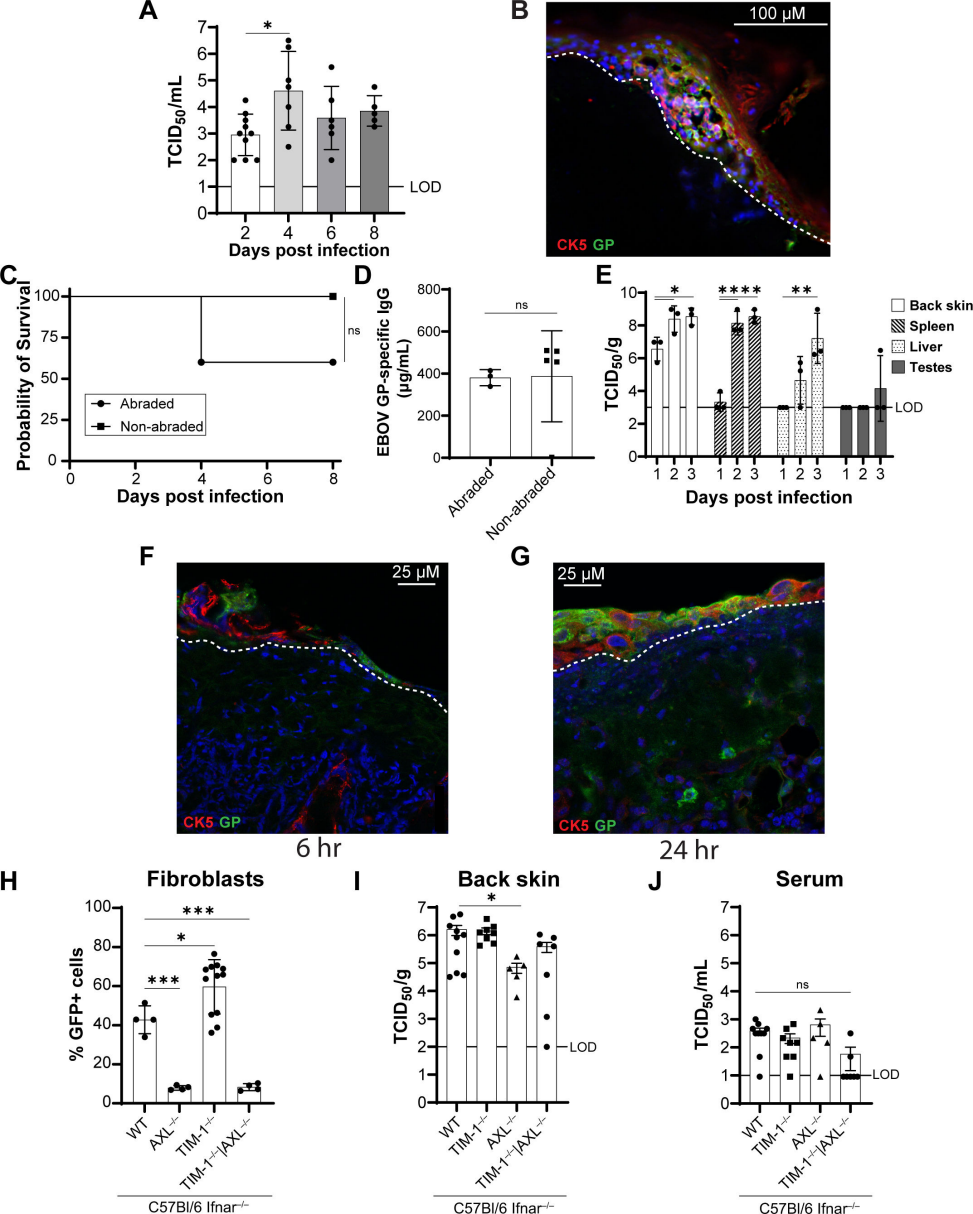
Infection of the epidermal surface of skin results in viral dissemination and morbidity. (**A and B**) NHP skin explants (*n* = 5–10, 1 cm^2^) were infected apically with rVSV/EBOV GP (1.5 × 10^7^ TCID_50_ in 1.5 µL DPBS). Infectious virus was measured in basal supernatants over time on Vero E6 cells (**A**). Explants were fixed on day 8 and stained for EBOV GP (green), CK5 (red), and DAPI (blue) (**B**). (**C and D**) C57BL/6 *Ifnar^–/–^* male mice (6−8 weeks old, *n* = 5 per group) were infected topically with rVSV/EBOV GP (10^9^ TCID_50_ in 10 µL DPBS) on either abraded or non-abraded back skin. Survival (**C**) and serum anti-EBOV GP IgG titers at 21 dpi (**D**). Significance was determined by the log-rank test (**C**) or Student’s *t* test (**D**). (**E**) Viral titers in tissues following topical application of rVSV/EBOV GP (10^7^ TCID_50_ in 10 µL DPBS) to abraded skin. Tissues were harvested at the indicated time points, and titers were determined on Vero E6 cells (*n* = 3). (**F and G**) Immunostaining EBOV GP (green), CK5 (red), and DAPI (blue) of skin at the inoculation site at 6 (**F**) and 24 (**G**) hours following topical administration of 10^7^ TCID_50_ rVSV/EBOV GP to abraded skin. The dotted line indicates the epidermal-dermal junction. Brightness and contrast were adjusted uniformly across the entire image for visualization purposes. Scale bar (μm) is shown in each panel. (**H**) Primary fibroblasts (*n* = 4–12 from 1 to 3 mice) were isolated from WT, *TIM-1^–/–^*, *AXL^–/–^*, or *Tim-1^–/–^* | *AXL ^–/–^* C57BL/6 *Ifnar ^–/–^* skin. Cells were infected with rVSV/EBOV GP at a multiplicity of infection (MOI) of 10, and GFP was measured in virally infected cells by flow cytometry at 48 hours post-infection. Data are expressed as mean ± SD. A one-way analysis of variance (ANOVA) was used to determine statistical significance, *P < 0.05; ***P < 0.001. (**I and J**) Six- to 10-week-old receptor KO and WT C57BL/6 *Ifnar^–/–^* mice (*n* = 5–8) were infected topically with rVSV/EBOV GP (10^9^ TCID_50_ in 10 µL DPBS) on abraded skin. Viral titers were measured in back skin (**I**) and serum (**J**) at 24 hours post-infection. Data are shown on a log_10_ scale as geometric means ± geometric SD. A one-way ANOVA on transformed values was used to determine statistical significance for each tissue (*P < 0.05; **P < 0.01; ***P < 0.001; ****P < 0.0001).

For NHP skin explants, 1.5 × 10^7^ TCID_50_ in 1.5 µL DPBS was applied to the skin surface and allowed to absorb. To monitor viral transit from the skin’s surface through the explant, infectious virus was measured in the basal medium over 8 days of infection. Infectious rVSV/EBOV GP was evident in basal media at 2 days post-infection, with significant increases by day 4 (Fig. 5A). At day 8, large foci of viral antigen were observed in the epidermis of FFPE sections (Fig. 5B; Fig. S9A), indicating that NHP keratinocytes readily support EBOV-GP-mediated infection, similar to human keratinocytes (9).

To assess the *in vivo* dissemination of topical virus application, the virus (10^9^ TCID_50_ rVSV/EBOV GP) was applied to the surface of non-abraded or abraded skin and survival monitored (Fig. 5C). All skin in these studies had been shaved 5 days prior to the experiments to remove the fur at the site of infection. To abrade skin, an emery board was used to remove the stratum corneum. Antibody titers were measured at 21 days in surviving mice (Fig. 5D). Removal of the stratum corneum and administration of high-titer viral inocula to mouse skin caused weight loss, morbidity, and mortality (Fig. S9B and C). Forty percent of the mice succumbed after being dosed topically with a high viral inoculum, while 100% of the mice without abrasion survived (Fig. 5C). Interestingly, abrasion was not necessary to stimulate robust EBOV-GP-specific IgG antibody titers (Fig. 5D). Additionally, administration of lower viral doses to abraded skin also stimulated robust IgG response in serum at 21 days post-infection (Fig. S9D).

To test whether antibody responses could be elicited in IFNAR-competent, WT C57Bl/6 mice, rVSV/EBOV GP was inoculated on abraded or non-abraded skin. At 21 days, abraded mice had a wide range of anti-EBOV GP IgG titers, with three of five mice surpassing the protective threshold of 100 µg/mL (40), while levels of non-abraded mice clustered between 30 and 60 µg/mL, a range associated with only partial protection (Fig. S9E).

After virus administration to abraded skin, infectious titers were assessed in various tissues. The shaved back skin (infection site) exhibited high viral titers by day 1 post-infection, which increased by day 2 (Fig. 5E). Evidence of systemic infection was provided by robust infection of the spleen by day 2 and the liver by day 3. The virus reached the testes in a single mouse by day 3.

To visualize viral spread at the site of infection in the skin, mice were euthanized at 6 and 24 hours post-infection. At 6 hours, viral antigen was restricted to a small focus in the epidermis (Fig. 5F). By 24 hours, infection had spread within the epidermis and reached myeloid-like cells in the deeper dermis (Fig. 5G). In contrast, infection of mice with non-abraded skin revealed high titers in the back skin, but little to no infectious virus was found in the spleen, liver, and serum (Fig. S9J). Secondary control and uninfected tissue sections confirming specific staining and single-channel images with colocalization analysis for Fig. 5 are shown in Fig. S9.

These experiments demonstrate that primary skin infection with rVSV/EBOV GP can infect and disseminate through the skin, supporting the possibility of skin-mediated EBOV transmission, which may require or be enhanced by breaks in the stratum corneum.

### Host receptors critical for topical rVSV/EBOV GP infection

Several cell surface receptors have been identified that enhance EBOV or rVSV/EBOV GP adherence and internalization into the endosomal compartment, where EBOV GP interacts with its cognate receptor, NPC1 (37, 41–46). Phosphatidylserine (PS) receptors and C-type lectins have been implicated as important receptors in tissue culture studies (41, 43, 44, 46). Using PS on its envelope, EBOV virions can bind to several different PS receptors, including TIM or TAM receptors, which mediate adherence and endocytosis. To date, only the PS receptor and TIM-1 have been shown to contribute to infection *in vivo*. At late times during infection, TIM-1 KO mice have lower rVSV/EBOV GP, but not rVSV/G, titers and reduced viral pathogenesis when administered IV (37). While AXL, a TAM receptor, is critical for EBOV entry into primary and immortalized keratinocytes and fibroblasts (9), the role of this cell surface receptor in systemic infection has not been evaluated.

To verify the host cell surface receptors that are required for rVSV/EBOV GP infection of cells derived from mouse skin, we isolated primary fibroblasts from the skin of *Ifnar^–/–^* mice and *Ifnar^–/–^* mice that have whole-body knockouts of either *Havcr-1* (that encodes TIM-1), *AXL*, or a combination of these (*Havcr1^–/–^* | *AXL^–/–^*). Primary fibroblasts were infected with rVSV/EBOV GP (MOI = 10) and evaluated for virus-encoded GFP expression by flow cytometry (Fig. 5H). Loss of AXL, but not TIM-1, significantly reduced the number of virus-infected cells.

We next sought to determine which host cell receptors are required for rVSV/EBOV GP infection of and dissemination from skin. We infected shaved and abraded skin on *Ifnar^–/–^*, *AXL^–/–^ Ifnar^–/–^*, and *Havcr1^–/–^ Ifnar^–/–^* mice and necropsied mice 24 hours after infection. Tissues from the back skin, spleen, serum, and liver were analyzed for infectious titers (Fig. 5I and J; Fig. S9K and L). The lowest titers in the back skin (site of infection) were found in *AXL^–/–^* mice, indicating that AXL is important for direct infection of the skin (Fig. 5I). However, titers in serum and spleen of *AXL^–/–^* and *Havcr1^–/–^* mice were not distinguishable from those found in *Ifnar^–/–^* mice, but there was an increase in infectious virus present in the liver of *Havcr1^–/–^* mice (Fig. 5J; Fig. S9K and L). Together, these data suggest that AXL impacts virus titers in the skin after inoculation, but the role AXL plays in systemic distribution of virus from the skin needs further evaluation.

## DISCUSSION

Studies of recent filovirus outbreaks have given a new appreciation for the role of skin in EBOV replication and pathogenesis. For instance, the detection of infectious virus and RNA on the skin surface provides a sensitive assay to assess EBOV infection status in EBOV treatment units (7). Our previous study identified cells within human skin explants that can support EBOV infection (9). Here, we define the kinetics and key cell types that support skin infection in NHPs and mice and identify several shared features of skin infection. In our models, viral loads increased in the skin over time, underscoring skin as a site of EBOV replication during systemic infection, particularly at late times of infection. EBOV antigen was abundant within the dermis of skin that was distal to the site of injection, providing evidence of trafficking to and active infection of dermal cell populations. Interestingly, we did not observe concomitant proinflammatory responses in mouse skin from either the thigh or back. This was in contrast to the proinflammatory responses found in the visceral tissues. Since skin is the largest organ in the body and serves as both a barrier and conduit to the external environment, recognition and appreciation of EBOV infection of this organ should help to guide the development of public control measures.

EBOV infects diverse skin cell types. We consistently observed viral antigen in CD45^+^ and CD45^−^ cells, highlighting that hematopoietic and non-hematopoietic cells contribute to skin infection (9). While endothelial cell involvement was observed in NHP skin tissue, viral antigen co-staining of murine skin endothelial cells was not observed. Previous reports indicate minimal endothelial infection outside the liver and lymph nodes in ma-EBOV-infected mice, suggesting model-dependent differences in infection (47). Viral antigen was detected in cells surrounding CD31^+^ cells, potentially implicating pericytes in infection. A prior study implicated EBOV virus-like particle-elicited alterations in pericyte cytokine production in the breakdown of the endothelial barrier (48). Keratinocyte infection in the epidermis was evident in *Ifnar^–/–^* rVSV/EBOV GP-infected mice, but the infection of these cells was limited in the EBOV-infected NHP or ma-EBOV-infected mouse epidermis. In human skin explants, extensive keratinocyte infection was noted (9), so the limited evidence of infection in our *in vivo* samples was unexpected. Our combined data suggest that while keratinocytes are permissive, virus access to these cells may be frequently delayed or restricted during *in vivo* infection. In addition, since we found that the skin infection was patchy, our immunostaining studies may have missed areas of robust epithelial staining, despite the large number of sections analyzed.

Abundant EBOV antigen was observed within and around hair follicles in all our models. This included CK5^+^ cells lining the follicle, CK5^−^ cells adjacent to the follicle lining, and cells within sebaceous glands. As an infectious virus can be detected on the surface of skin from EBOV-infected postmortem NHPs (8, 16), these data and the absence of robust *in vivo* epidermal infection suggest that EBOV secretion into hair follicles may be a mechanism for virus egress to the epidermal surface.

EBOV stimulates proinflammatory gene expression in several key tissues during ma-EBOV infection (21, 33, 49, 50). We found upregulated proinflammatory gene transcripts in visceral tissues. While some proinflammatory gene transcripts, such as ISG15 or IL-6, are likely derived from a wide range of hematopoietic and non-hematopoietic cells, elevated IFN-γ transcripts are likely produced by tissue lymphocytes, NK cells, and/or perhaps macrophages present in visceral tissues (17, 18, 24, 51, 52). The well-established murine immunomodulatory gene, Fizz-1, remained unchanged or was depressed in all tissues, whereas the second immunomodulatory gene tested, Arg1, was elevated in some tissues. This is likely due to STAT3-stimulated Arg1 expression, which is thought to be in response to proinflammatory stimulation (36).

Despite significant increases in viral load in the thigh skin, no significant changes in proinflammatory gene transcripts were detected in either the thigh skin or the back skin, suggesting limited innate immune responses to the infection in these tissues. The absence of significant immune responses to EBOV contrasts with a recent report of significant proinflammatory responses to EBOV infection in skin from Jamaican fruit bats (53). The patchiness of skin infection is responsible for the limited immune responses observed because the evaluated skin RNA samples were not taken from “hot spots” of infection but from randomly sampled skin. Future studies assessing focal areas of infection in skin should provide additional insights into both the mechanisms driving the focal nature of skin infection and whether immune responses occur or are suppressed within those “hot spots,” including both innate and adaptive components.

Using rVSV/EBOV GP, we directly tested whether the virus could topically infect skin and if the virus would disseminate from skin to systemically establish infection in *Ifnar^–/–^* mice. Removal of the stratum corneum by abrasion led to higher morbidity as evidenced by weight loss, modestly lower survival, and elevated infectious titers in key organs. In contrast, virus painted onto non-abraded skin of *Ifnar^–/–^* mice caused no overt disease, though all but one mouse generated high EBOV-GP-specific IgG titers. Topical administration of rVSV/EBOV GP to non-abraded skin of immunocompetent mice resulted in modest, sub-protective antibody levels. While abraded mice exhibited more variable anti-EBOV GP IgG levels, on average, these mice generated titers that would be predicted to protect against subsequent EBOV challenge (40). These data suggest that breaching the epidermal barrier not only facilitates systemic virus infection but also enhances antigen presentation and humoral immune activation. Indeed, prior studies have shown that antigens on intact skin can produce autonomous antibodies and restrict both local and systemic bacterial spread (54–56). Additional work has shown that B cells reside in healthy skin and that bacterial colonization of dermal tissue results in robust antibody responses (57, 58), supporting the potential of skin as a viable route for EBOV and other vaccinations (59, 60).

AXL is a cell surface PS receptor that mediates EBOV adherence and internalization into primary and immortalized human dermal fibroblasts and keratinocytes (9). The PS receptor, TIM-1, facilitates EBOV-GP-mediated entry into damaged kidney tubular cells, lung epithelial cells, and various epithelial cell lines (9, 37, 43, 44, 61, 62). Mice lacking AXL expression had reduced skin infection when the virus was directly applied to the skin. However, these mice did not have reduced systemic viral dissemination from skin. Hence, those cell surface receptors important for systemic EBOV dissemination from the skin remain to be determined. Together, our results underscore the significance of the skin as a primary site of EBOV infection and dissemination, thereby reinforcing its crucial role in the systemic pathogenesis of EBOV.

## MATERIALS AND METHODS

### Generation and titering of virus stocks

#### EBOV stocks at Texas Biomedical Research Institute

EBOV (Kikwit-9510621 P2) was obtained from Dr. Tom Ksiazek (at NIAID’s WRCEVA at UTMB’s Health Galveston National Laboratory) in 2012 and propagated at Texas Biomedical Research Institute. The stock virus used was a third passage virus propagated in Vero E6 cells and had a titer of 2.1 × 10^5^ PFU/mL as determined by an infectious plaque assay on Vero E6 cells as previously described (10). The stock has been confirmed to be wild-type Ebola virus by deep sequencing.

#### EBOV stock at NEIDL

EBOV (Kikwit P4) was propagated in Vero E6 cells, and the sequence was confirmed by deep sequencing to be identical at the consensus level to GenBank KR063672.1 and utilized in the Boston University NHP study described in detail below. EBOV (Mayinga) was sucrose cushion purified and utilized in the Boston University *Ifnar^–/–^* mouse study described in detail below.

#### ma-EBOV stocks at NEIDL

ma-EBOV (Ebola virus, Mayinga, mouse-adapted EZ-76) virus was propagated on Vero-E6 cells in the presence of 2% fetal bovine serum (FBS). When the cytopathic effect (CPE) was evident, the culture supernatant was collected, cell debris was pelleted by centrifugation at 1,000 × *g* for 10 minutes, and the clarified supernatant containing virus was stored frozen in 1 mL aliquots at −80°C. Virus titer was determined as described in a previous report (63). Briefly, Vero E6 cells were infected with ma-EBOV for 1 hour and then overlayed with methylcellulose. After 3 days, the overlay was removed, and the cells were fixed in formalin and then stained with a virus-specific antibody. Foci of infection were counted and used to back-calculate the virus titer in FFUs per milliliter.

#### rVSV/G and rVSV/EBOV GP at the University of Iowa

The control virus used in the BSL-2 studies, rVSV/G, encodes the native VSV G and GFP. rVSV/EBOV GP and rVSV/G *in vivo* viral stocks were propagated on Vero E6 cells, initiating infection with a low MOI (~0.001). Supernatants were collected when CPE was evident. Viral supernatants were filtered through 0.45 µM filters and concentrated and purified by layering virus over a 25% sucrose/DPBS cushion and ultracentrifuging for 2 hours at 120,000 × *g*. Endotoxin-free reagents were used to reduce LPS contamination. Viral titers were determined in TCID_50_ assays where serial dilutions of rVSV/EBOV GP were added to 6–8 replicate wells per dilution containing 10,000 Vero E6 cells per well in a 96-well plate. Infected cells were incubated for 5 days, and the TCID_50_ was determined using the Reed and Muench method (64).

### *In vivo* studies

The BSL-4 NHP study at the NEIDL was approved by the Boston University IACUC and performed in accordance with approval number PROTO201900012. BSL-4 ma-EBOV murine studies were performed in accordance with protocol # 201900062, which was reviewed and approved by the IACUC at Boston University. BSL-4 work was conducted in accordance with all institutional, local, state, and federal regulations and guidelines for BSL-4 containment work.

Animal research and breeding performed at the University of Iowa (UI) were conducted in accordance with the Animal Welfare Act and the recommendations in the Guide for the Care and Use of Laboratory Animals of the National Institutes of Health (PHS Animal Welfare Assurance Number: D16-00009, A3021-01). All animal procedures were designed to minimize animal discomfort and were approved by the UI IACUC. BSL-2 rVSV/EBOV GP *in vivo* mouse studies were performed in accordance with the IACUC guidelines (protocols #1031280 and #4021280 Filovirus glycoprotein/cellular protein interactions and virus glycoprotein/cellular protein interactions).

### Non-human primate studies

NHP skin samples were obtained from independent EBOV infection studies performed at either the Texas Biomedical Research Institute or the NEIDL at Boston University. Samples include tissues from EBOV- or mock-infected rhesus macaques. In all studies, NHPs were acclimated for 7 days in the ABSL-4 before infection.

#### Texas Biomedical Research Institute: EBOV *in vivo* NHP time course infection

RNA samples were received at the University of Iowa from EBOV-infected NHPs euthanized over the course of infection as previously described (10). In this study, 20 male and female rhesus macaques, aged 3–7 years, were infected with 1,000 PFU of EBOV (Kikwit) IM, with four animals serving as mock-infected controls (sterile phosphate-buffered saline [PBS]). All animals were euthanized at scheduled times on days 3, 4, 5, and 6 of infection, or when the endpoint was reached at 7 or 9 dpi. Skin samples from the injection site were placed in TRIzol, and 19 of these samples were evaluated for viral load at the University of Iowa. Virus titers in other organs obtained from this study have been previously described (10).

#### Texas Biomedical Research Institute: EBOV *in vivo* NHP antiviral study (treatment control animals only)

In a second study, material was obtained from two animals (mixed sex, ~3 years of age, ~5 kg) used as infected treatment controls in a study evaluating antivirals for EBOV. Each received saline only as treatment. On the day of exposure, day 0, NHPs received an IM inoculation of 1,000 PFU of (P3) EBOV (Kikwit). Animals were observed at least twice daily for morbidity and mortality until the endpoint was reached (days 6 and 8). At the end point of euthanasia, a representative sample of skin, including the epidermis, dermis, and subcutis with underlying muscle, was collected from the site of inoculation and was placed in 10% buffered formalin for fixation for a minimum of 21 days. The sample was then paraffin-embedded and sectioned before being sent to the University of Iowa for immunostaining.

#### Boston University (NEIDL): EBOV *in vivo* NHP infections (control NHPs for antiviral study)

Rhesus macaques (two males and two females, 3.5–6 kg, aged 4 years) were infected IM in the right deltoid muscle with 100 PFU passage 4 EBOV (Kikwit) diluted into 0.5 mL of PBS. Following exposure, animals were observed at least twice daily, increasing to at least four times daily as the disease progressed. All animals were humanely euthanized at days 6 or 7 when they were determined to be moribund. Haired skin at and contralateral to the injection site was collected at necropsy and placed into 10% neutral buffered formalin for more than 72 hours at room temperature.

### Mouse studies

#### Mouse strains

WT C57Bl/6 mice were purchased from Jackson Lab (Bar Harbor, ME). C57BL/6J *Ifnar^–/–^* mice were a kind gift from Dr. John Harty (University of Iowa, Iowa City, IA, USA). All C57BL/6 *Ifnar^–/–^* lines were bred at the University of Iowa. C57BL/6 *Ifnar^–/–^* and *HAVCR1* (*TIM-1)^–/–^ Ifnar^–/–^* have been previously described (25, 37). C57BL/6 *AXL^–/–^* were obtained from Dr. Rolf Brekken (Univ. Texas, Southwestern, Houston, TX) and crossed onto a C57BL/6 *Ifnar^–/–^* background, generating C57BL/6 *AXL^–/–^ Ifnar^–/–^*. Triple knockout C56BL/6 *TIM-1^–/–^ AXL^–/–^ Ifnar^–/–^* (*TIM-1^–/–^* | *AXL^–/–^*) were generated by crossing C57BL/6 *TIM-1^–/–^ Ifnar^–/–^* with C57BL/6 *AXL^–/–^ Ifnar^–/–^*. Mouse genotypes were verified using primers described in Table 3 and PCR conditions previously described (37). Briefly, as approved and described by the UI IACUC policies and guidelines, a small <5 mm of the mouse tail is snipped and processed using the Wizard Genomic DNA Purification Kit by Promega as described by the manufacturer’s instructions. DNA is quantified by nanodrop. For each reaction, 100 ng of genomic DNA, primers, and the New England Biolabs OneTaq 2X Master Mix are mixed, and a touchdown PCR protocol is used as recommended by Jackson Labs.

**TABLE 3:** Primers used to genotype mice

Gene	Forward common primer (5′ → 3′)	Reverse mutant primer (5′ → 3′)	Reverse WT primer (5′ → 3′)
TIM-1	GTTTGCTGCCTTATTTGTGTCCTGG	GTCTGTCCTAGCTTCCTCACTG	GTCTGTCCTAGCTTCCTCACTG
AXL	ATCTGTCCCCCATACCCTCTGACA	GGGCCAGCTCATTCCTCCCACTCAT	TTCCATGACTTCAGCTTCCCCGATG
Ifnar	ACTCAGGTTCGCTCCATCAG	GAACCTGAGGCTGTCGAAGG	CTTTTAACCACTTCGCCTCGT

#### Boston University (NEIDL): ma-EBOV *in vivo* infections

Fifty 6-week-old, female C57BL/6N mice were infected IP with 1,000 FFU of ma-EBOV (65). Animals (*n* = 10) were euthanized every 24 hours from days 1 to 5 of infection. Tissues collected at specified time points were liver, subcutaneous fat from the belly, visceral fat in the belly region, belly skin (adjacent to the injection site), back skin (distal to the site of injection), and inner thigh skin (distal to the site of injection). The tissue was either processed using TRIzol Reagent to measure transcriptional changes or fixed in formalin for 72 hours and then paraffin-embedded for immunostaining studies, as described in detail below.

#### Boston University (NEIDL): EBOV *in vivo Ifnar ^–/–^* mouse infections (untreated control mice for antiviral study)

Five C57BL/6 *Ifnar^–/–^* mice were infected IP with 1,000 FFU of EBOV (Mayinga). Animals were euthanized when endpoint criteria were met. The back skin (distal to the injection site) was fixed in formalin for 72 hours and then paraffin-embedded for immunostaining studies, as described in detail below.

#### rVSV/EBOV GP *in vivo* IP infections (inside-out IP infections)

Serial dilutions of each rVSV/EBOV GP stock were injected IP or painted on shaved, abraded skin of *Ifnar^–/–^* mice to determine the appropriate viral dose used in the studies. A similar dilution of rVSV/G stock was injected IP to determine the dose needed to achieve equivalent lethality to rVSV/EBOV GP. Weight loss, morbidity, and mortality were measured to assess the LD of each stock generated per route of infection.

To investigate EBOV-GP-mediated infection to distal skin, 6–10 week old, male mice on a C57BL/6 *Ifnar^–/–^* background were shaved on the right back flank (an area approximately 2 × 2 cm^2^) and subsequently infected IP with 500 TCID_50_ of rVSV/EBOV GP (~LD_100_ within 3–5 days) or 1,000 TCID_50_ of rVSV/G (~LD_100_ within 3–5 days) which served as a control. Specific mouse genotypes, time points, and number of mice can be found in the figure legends.

Mice were euthanized from 1 to 3 dpi, and the spleen, liver, and back skin were collected. In some experiments, serum, cheek, and belly skin samples were also collected. Serum was collected by bleeding mice using a Goldenrod Animal Lancet (5 mm, SKU: GR 5 MM, MedPoint) to obtain blood from the submandibular vein. Blood was collected in a microtainer blood collection tube (BD SST, Gold, CAT: 365967) and spun at 10,000 × g for 10 minutes. Serum was collected, placed in an Eppendorf tube, and kept at −80°C for viral titering as described below. Samples of the back and cheek skin were also obtained and fixed in formalin for 24 hours and later paraffin-embedded for immunostaining. For titering, all tissues were weighed before homogenization in 0.5 mL DPBS using 1.5 mL Eppendorf pestles. Tissues were stored at −80°C until viral titers were determined. Tissues weighing below the scale’s detection limit were assigned a weight of 0.004 g.

Some skin tissues were separated into epidermal and dermal tissue before titering. Five 3 mm punch biopsies were taken from the shaved back and submerged into 5 mg/mL of dispase II (Millipore Sigma, #D4693) solution (diluted in DPBS) for 2 hours at 37°C. The epidermis was separated from the dermis using forceps. The epidermis and dermis per mouse were weighed and homogenized in 0.5 mL DPBS. All tissue was placed at −80°C until titered.

Infectious virus trafficking to the apical surface of the back skin in mice was also measured. One hundred microliters of 1× DPBS was placed on the surface of shaved back skin for two minutes before the wash was collected and placed at −80°C until titered.

Focal infections were visualized by shaving a large region of back skin (~5 × 5 cm^2^) of 6–10-week-old, C57BL/6 *Ifnar^–/–^* male mice. Mice were held for 5 days to allow the stratum corneum to be regenerated. Shaved mice were infected IP with a lethal dose of rVSV/EBOV GP. Mice were euthanized when endpoint criteria were met (3 dpi). Serum, spleen, and liver were collected as described above. Shaved back skin was harvested, placed on a microscope slide, and visualized by a fluorescence laser on a Leica M165 FC Fluorescent Stereo Microscope. To capture the focal infection, images of the skin mounted on the fluorescence stereo microscope stage were taken in the dark with a Nikon D3100 digital camera using a Tamron SP 90 mm f/2.8 Di 1:1 AF Macro Lens. To obtain the images of the visible fluorescence signal in the skin, the 405 nm laser was turned on, and images were taken through a UV filter shield with the following acquisition settings: f/4.5–5.8 f-stop, 1/125-second exposure time, maximum aperture between 4.3 and 5, ISO speed 3,200, exposure bias of 0 steps, and no flash. Images were processed using ImageJ software to subtract the background and add scale bars. Dermal biopsy punches (3 mm) were taken from several areas where GFP was and was not evident. Titering was carried out as described above, or biopsies were further dissociated and processed for flow cytometry. The epidermis was separated from the dermis as described above. The dermis was further processed in 100 µg/mL of DNase I (Millipore Sigma, Product number: 10104159001) and 1 mg/mL of Collagenase P (Millipore Sigma, Product number: COLLP-RO) solution (diluted in Dulbecco’s modified Eagle medium [DMEM]/high glucose) for 1 hour at 37°C. The epidermis was dissociated in pre-warmed 0.05% trypsin/EDTA at 37°C for 10 minutes. Dissociated tissues were filtered through 100 µm cell strainers and washed with 1× DPBS. Cells were centrifuged at 700 × *g* for 5 minutes and resuspended in Flow Cytometry buffer (1× DPBS, 2% FBS, and sodium azide). Samples were measured for GFP^+^ on a CytoFLEX cytometer (Beckman Coulter, Brea, CA, USA). Data were analyzed using FlowJo software version 10.10.0 (BD Biosciences, Franklin Lakes, NJ, USA).

#### rVSV/EBOV GP *in vivo* skin infections (outside-in)

To investigate EBOV-GP-mediated viral dissemination from topical skin infection, 6–10-week-old, male mice on a C57BL/6J *Ifnar^–/–^* background were shaved on the right back flank. Five days after shaving, the shaved skin of some mice was gently abraded with an emery board until shiny (abraded). A 10 µL aliquot of concentrated virus was painted on the skin surface of abraded and non-abraded mice. Mice were monitored until the virus dried. Details of specific mouse genotypes, time points, and numbers can be found in the figure legends.

Weight loss, morbidity, and mortality were monitored throughout the infection. Surviving mice were kept for 21 days and then bled by puncturing the submandibular vein with a Goldenrod animal lancet. Blood was collected in a microtainer blood collection tube (BD SST, Gold) and spun at 10,000 × *g* for 10 minutes. Serum was collected, placed in an Eppendorf tube, and stored at 4°C to measure EBOV-GP-specific serum IgG antibody titers as described below.

Mice were euthanized from 6 hours to 3 days post-abraded skin inoculation, and the spleen, liver, back skin, and testes were collected. Samples of the back were obtained and fixed in formalin for 24 hours and later paraffin-embedded for immunostaining.

### NHP explant studies

#### NHP explant infection

NHP skin explants were generated from rhesus macaque skin samples (*n* = 2 macaques). The skin tissue was made available postmortem from animals on a per-cost basis from the Wisconsin National Primate Research Center. A 1 cm² explant was placed on semipermeable transwell inserts (2.3 cm diameter, 3.0 µM pore size; Falcon 353091). Explants were maintained up to 12 days post-infection in 1.5 mL of minimum essential medium (MEM) supplemented with 10% FBS, 100 units/mL of penicillin, 100 µg/mL of streptomycin, amphotericin B, and 0.2 µg/mL of type I interferon (IFN) inhibitor B18R. Explants were infected with 1.5 × 10^7^ TCID_50_ of rVSV/EBOV GP added to the basal media or placed on the apical epidermal surface in 1.5 µL and allowed to dry in the biological safety cabinet before the plate was returned to the incubator. Following overnight incubation, explants were washed twice with DPBS, and the media were replaced. Basal supernatants were collected to measure viral infection on Vero E6 cells by TCID_50_, and the media were replaced every other day. Some tissues were fixed for immunostaining on day 8 post-infection in 4% paraformaldehyde overnight at 4°C.

### Assays detecting virus infection and replication

#### TCID_50_ studies

Vero E6 cells were plated at 10,000 cells per well in a 96-well format. The following day, homogenized tissues and serum samples were allowed to thaw to room temperature and filtered through 0.45 µM filters. Serial dilutions of samples were added to 6–8 replicate wells of cells. The virus was incubated for 5 days, and the TCID_50_ was determined using the Reed and Muench method, expressed as TCID50 per gram or milliliter. All tissues underwent a single freeze-thaw.

#### RT-qPCR

ma-EBOV tissue was homogenized in TRIzol LS using a Qiagen TissueLyzer with a 5 mm stainless steel bead according to the manufacturer’s directions, running two cycles, 2 min each at 30 Hz. Samples were then clarified by centrifugation, frozen at −80°C, and shipped to the University of Iowa after being certified that all the virus was inactivated. Sample RNA was extracted following the manufacturer’s instructions. Total RNA was quantified using a Nanodrop Spectrophotometer (ND-1000) to determine the sample volume required to achieve 1 µg of RNA. cDNA was generated using the High-Capacity cDNA Reverse Transcriptase kit (Applied Biosystems, Cat: 4368814) as described by the manufacturer’s instructions. Using PowerUP SYBR Green master mix (Applied Biosystems, Cat: A25743), quantitative PCR was performed on QuantStudio 3 Real-time PCR machine (Applied Biosystems) to calculate the ΔCT values in relation to the housekeeping gene. Specific primer sequences are listed in Table 4.

**TABLE 4:** Oligonucleotides used in this study

Gene	Forward primer (5′ → 3′)	Reverse primer (5′ → 3′)
EBOV VP40	CATTTCGGCAAGGCAACCAA	AGTTGGACTGGCGGAAGAAC
EBOV L	TGCGCCAGATTGTACGCAGGA	CGCTCGGCGTGCGTGAAAAG
NHP GAPDH	ACAACAGCCTCAAGATCGTCAG	ACTGTGGTCATGAGTCCTTCC
Mouse CypA	GCTGGACCAAACACAAACGG	ATGCTTGCCATCCAGCCATT
Mouse IFN-γ	CGGCACAGTCATTGAAAGCC	TGTCACCATCCTTTTGCCAGT
Mouse ISG15	TCCTGGTGAGGAACAAAAGG	CGCAAATGCTTGATCACTGT
Mouse Fizz1	ACTTCTTGCCAATCCAGCTAAC	CAAGCACACCCAGTAGCAGT
Mouse Arg1	CAAATTGTGAAGAACCCACGG	CTTCCAACTGCCAGACTGTG

### Anti-EBOV GP IgG ELISAs

Anti-EBOV GP antibodies were detected using an enzyme-linked immunosorbent assay (ELISA). This was performed by coating optical microtiter plates (Immulon HB) overnight with soluble EBOV GP (Kikwit-95, Acro Biosystems; 50 µL/well at 1 µg/mL). A standard curve of mouse IgG was generated and plated in duplicate in non-coated wells (Southern Biotech, Cat: 0107-01). Wells were washed four times with ELISA Wash Buffer (1× DPBS + 0.015% Tween 20), blocked for 2 hours at 25°C with ELISA Blocking Buffer (1× DPBS + 2% bovine serum albumin [BSA]; 200 µL/well) and incubated overnight at 4°C with one of three serum dilutions (1:1,000, 1:10,000, and 1:100,000) in duplicate obtained from mice surviving rVSV/EBOV GP infections. Following incubation, the wells were washed as described above and then incubated with horseradish peroxidase (HRP)-conjugated anti-mouse IgG (in 50 µL of ELISA blocking buffer; 1:500 dilution of antibody) for 1 hour at 25°C. Wells were washed as described and incubated with HRP substrate according to the manufacturer’s protocol (TMB Substrate, BD Biosciences, Cat: 555214). The reaction was stopped with 50 µL 2M H_2_SO_4_ , and the absorbance at 450 nm was measured on a Tecan Plate Reader (Life Sciences). Total concentration of anti-EBOV GP antibodies was quantified by comparison to the standard curve.

### Immunofluorescence and H&E staining of animal tissues

#### Immunofluorescence

Direct immunofluorescence staining was performed on 7 µM FFPE tissue sections. Tissue sections were deparaffinized by immersing the slides in xylene and then rehydrated through a graded ethanol series (100%, 95%, 70%, and 50%). The slides were rinsed in ddH_2_O, and epitope retrieval was performed by incubating the slides in pre-warmed 10 mM citrate buffer (pH 6.0), followed by microwaving at 90°C for 5 minutes. After cooling for 5 minutes, the heating step was repeated. Sections were subsequently washed in ddH_2_O and permeabilized with 0.25% Triton X-100 diluted in TBS Ca+/azide staining buffer. To block non-specific binding, slides were incubated with 5% animal serum. After blocking, primary and secondary detection antibodies were applied. The sources, optimal concentrations, and experimental conditions used for staining with each antibody are presented in Table 5. Slides were dipped in ddH_2_O to remove salts and then mounted with DAPI mounting medium (Abcam/ab104139). Images were collected using a Leica SPE confocal microscope. In some cases, color adjustments were applied to whole images, or images were cropped using ImageJ and Adobe Photoshop software.

**TABLE 5:** Antibodies used in immunostaining studies

Antibody description	Vendor/Item ID	Host species	Dilution	Incubation conditions
EBOV GP	IBT Bioservices/0260-001	Human	1:100	Overnight/4°C
EBOV VP40	IBT Bioservices/0201-016	Mouse	1:100	Overnight/4°C
EBOV VP40	IBT Bioservices/0301-010	Rabbit	1:100	Overnight/4°C
ASGR1	Proteintech/11739-1-AP	Rabbit	1:50	Overnight/4°C
CD31	Invitrogen/PA5-16301	Rabbit	1:100	2 h/RT
Cytokeratin 5	Invitrogen/PA5-29670	Rabbit	1:200	2 h/RT
CD3ε	Abcam/ab5690	Rabbit	1:300	Overnight/4°C
CD11b Biotin	TONBO Biosciences/30-0112-u025	Rat	1:100	2 h/37°C
IBA1	Fujifilm/019-19741	Rabbit	1:300	2 h/RT
MHC Class II	Invitrogen/14-5321-82	Rat	1:100	2 h/RT
CD45 Monoclonal Antibody	Invitrogen/14-0451-85	Rat	1:100	2 h/RT
PLIN1	Abcam/ab3526	Rabbit	1:200	Overnight/4°C
F4/80	Cell signaling/D4C8V	Rabbit	1:100	2 h/RT
Goat anti-Rabbit IgG, Alexa Fluor Plus 555	Invitrogen/A-48283	Goat	1:800	1 h/RT
Streptavidin, Alexa Fluor 555 Conjugate	Invitrogen/S32355	N/A^*a*^	1:500	1 h/37°C
Goat anti-Rabbit IgG, Alexa Fluor 488	Invitrogen/A-11008	Goat	1:800	1 h/RT
Goat anti-Human IgG, Alexa Fluor 647	Jaksonimmuno/109-605-003	Goat	1:1,000	1 hr/RT
Goat anti-Rat IgG, Alexa Fluor™ 555	Invitrogen/A21434	Goat	1:800	1 h/RT
Goat anti-Mouse IgG, Alexa Fluor 488	Invitrogen/A-11029	Goat	1:800	1 h/RT

^*a*^ N/A indicates not applicable.

#### Viral antigen percent area quantification

The percent area positive for viral antigen was quantified using ImageJ (NIH). Unedited immunofluorescence images were separated into individual channels, and the green channel (viral antigen staining) was selected for analysis. Thresholding was applied using the *Adjust Threshold* function to highlight areas of positive staining while excluding background. Threshold values were determined using both positive and negative controls and applied uniformly across all samples. The maximum threshold was set to 255. Regions of interest (ROIs) were manually defined to exclude scale bars. Area fraction measurements (% area positive) were recorded using the *Measure* function, with additional outputs including area, limit to threshold, and display label.

#### Colocalization analysis

Colocalization of viral antigen with cell-specific markers was assessed using the “Colocalization Highlighter” plugin from the MBF ImageJ plugin collection (https://imagej.net/ij/plugins/mbf/index.html). After channel separation, threshold values for both the green (viral antigen) and red (cell marker) channels were determined based on control images and applied consistently across all images. The Colocalization Highlighter was used to visualize overlapping signals, with colocalized pixels rendered in white. The resulting composite image was split into individual channels, and the colocalization signal was isolated in the blue channel. ROIs were defined as above, and the percent area of colocalization was quantified in the blue channel. Specificity of colocalization was validated by rotating one channel 90°, which abolished the signal.

#### H&E staining

H&E staining was performed at the Comparative Pathology Core (University of Iowa). FFPE tissues were dehydrated through a progressive series of alcohols and xylene, paraffin-embedded into blocks, and sectioned (~4–5 µm) onto glass slides. The tissues were stained with an H&E stain. A boarded veterinary pathologist examined tissues.

### Primary murine fibroblast studies

#### Generation and infection of primary murine fibroblast cultures

Mice were necropsied, and their skin was dissected and then passed through 70% ethanol and povidone-iodine solutions to sterilize, followed by several DPBS washes. Small sections of mouse skin tissue were embedded with a scalpel into the plastic of 10 cm^2^ tissue culture treated plates. Tissue was maintained in DMEM with 10% FBS and 100 units/mL of Penicillin and 100 µg/mL of Streptomycin and replaced weekly. Within 2–3 weeks, fibroblasts grew out from the skin sections, creating a monolayer. Cells were passaged one or two times before infection experiments.

All primary fibroblasts were cultured in triplicate in a 48-well plate at ~100,000 cells per well in a volume of 400 µL of DMEM with 10% FBS and 100 units/mL of Penicillin and 100 µg/mL of Streptomycin. Fibroblasts were infected for 48 hours at an MOI of 10 with rVSV/EBOV GP. Cells were detached with 0.25% trypsin/EDTA, neutralized with an equivalent volume of newborn calf serum, centrifuged, and resuspended in DPBS with 2% calf serum with azide. Cells were analyzed by flow cytometry on Beckman CytoFLEX to quantify the frequency of GFP^+^ cells.

### Statistical analysis

Statistical analysis was performed in Prism. Graphs on a log scale had statistics performed on log-transformed values. Statistical tests included Student’s *t* test, Mann-Whitney *U* test, and a one-way analysis of variance. Flow cytometry analysis was done in FlowJo. More specific details regarding statistical tests and exact *n* per group can be found in the figure legends.

## Data Availability

Raw RT-qPCR data shown in Fig. 1 to 3 and Fig. S4 and 7 are available upon request. Antibodies used in immunofluorescence staining are detailed in Table 5, with catalog numbers and staining conditions included.

## SUPPLEMENTAL MATERIAL

Supplemental figures jvi.01300-25-s0001.docx Figures S1 to S9.

ASM does not own the copyrights to Supplemental Material that may be linked to, or accessed through, an article. The authors have granted ASM a non-exclusive, world-wide license to publish the Supplemental Material files. Please contact the corresponding author directly for reuse.
