# Supplementary material for: Ebola virus’ hidden target: virus transmission to and infection of skin
Source: J Virol. 2025 Sep 12;99(10):e01300-25. doi: 10.1128/jvi.01300-25 (PMC12548420; doi:10.1128/jvi.01300-25)
Supplement: Supplemental figures — Figures S1 to S9. [file jvi.01300-25-s0001.docx]

Supplementary Materials for **Ebola Virus’ Hidden Target: Virus Transmission to and Infection of Skin**

Paige T. Richards *et al.*

*Corresponding author. Email: wendy-maury@uiowa.edu

**This PDF file includes:**

**Table of content for Supplementary Materials**

Fig. S1. EBOV traffics to and infects NHP skin during systemic infection: staining controls of NHP skin tissues, additional images, and rVSV/EBOV GP infection of explants.

Fig. S2. EBOV traffics to and infects NHP skin during systemic infection: single channel images and colocalization analyses for Figure 1.

Fig. S3. EBOV traffics to and infects NHP skin during systemic infection: single-channel images and colocalization analyses for Supplemental Figure 1.

Fig. S4. ma-EBOV kinetics and viral cell tropism in mice: staining controls and additional images.

Fig. S5. ma-EBOV kinetics and viral cell tropism in mice: single channel images and colocalization analyses for thigh skin panels in Figure 2 and Supplemental Figure 4.

Fig. S6. ma-EBOV kinetics and viral cell tropism in mice: serial sectioning of distal back skin from EBOV-infected *Ifnar ^–/–^* mice.

Fig. S7. Inflammatory profiles of ma-EBOV-infected mouse tissues: additional tissue sites.

Fig. S8. rVSV/EBOV GP targets similar cells as ma-EBOV in distal skin: rVSV/G titers, staining controls, and focal virus distribution in skin.

Fig. S9. Infection of the epidermal surface of skin results in viral dissemination and morbidity: survival curve, IgG production and staining controls.


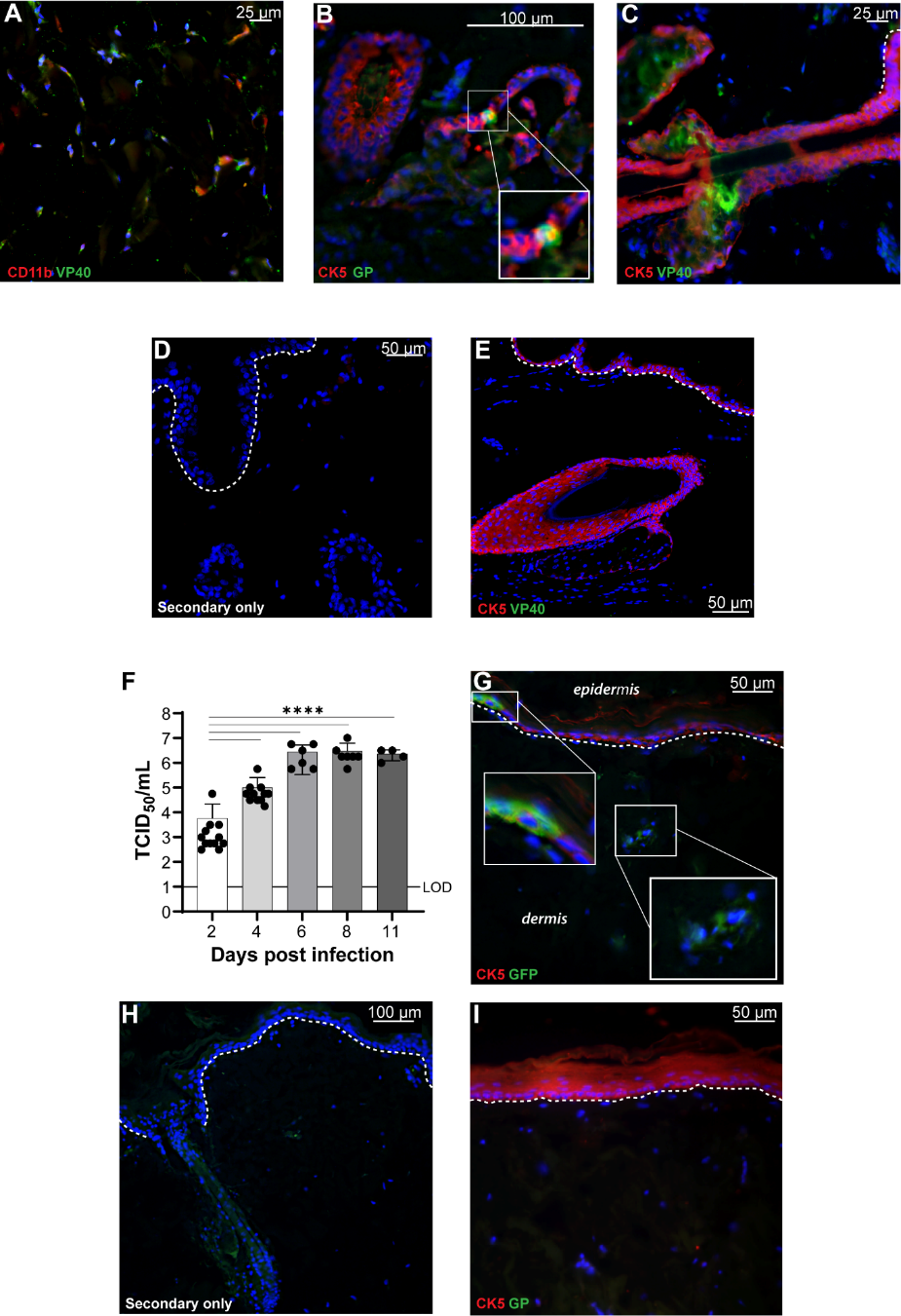


**Fig. S1. EBOV traffics to and infects NHP skin during systemic infection: staining controls of NHP skin tissues, additional images, and rVSV/EBOV GP infection of explants.** **A–C**) EBOV antigen colocalizes with cell-specific markers in NHP skin. **A**) EBOV antigen and CD11b colocalize in dermal cells from contralateral skin at 7 dpi. **B**) EBOV GP^+^ cells are detected in CK5+ hair follicle keratinocytes at the injection site on day 6. **C**) EBOV VP40^+^ cells are found within sebaceous glands near hair follicles in contralateral skin on day 7. Panel **A** is from the NEIDL cohort (100 PFU EBOV) described in Fig. 1, while panels **B** and **C** are from a separate study performed at Texas Biomed (1000 PFU EBOV). **D–E**) Immunostaining controls for Fig. 1: **D**) secondary antibody-only control of EBOV-infected NHP skin; **E**) mock-infected NHP skin stained with primary and secondary antibodies. **F–G)** rVSV/EBOV GP infects NHP skin explants. NHP skin explants (n=6 from two NHPs, 1 cm^2^, with some harvested for immunostaining) were infected by addition of rVSV/EBOV GP (1.5 x 10^7^ TCID_50_) in the basal media. **F**) Infectious virus was detected in basal media over 11 days post-infection. Data are shown from 2 independent experiments with data expressed on a log_10_ scale as means ±SEM. Statistical significance was determined by ordinary one-way ANOVA, (****p<0.0001). **G**) GFP^+^ viral antigen (green) detected in the dermis and colocalized with CK5^+^ (red) cells in the epidermis at 8 days post-infection. **H–I**) Staining controls for NHP skin explants with secondary antibodies alone (**H**) and primary and secondary antibodies in mock-infected skin explants (**I**). Brightness and contrast were adjusted uniformly across the entire image for visualization purposes. The dotted line in panels **A**, **B**, **D**, **F–H** denotes the location of the epidermal-dermal junction. Single channels and colocalization analyses for panels **C–E** and **G** are shown in Supplemental Fig. 3. Scale bar (μm) is shown in each panel.


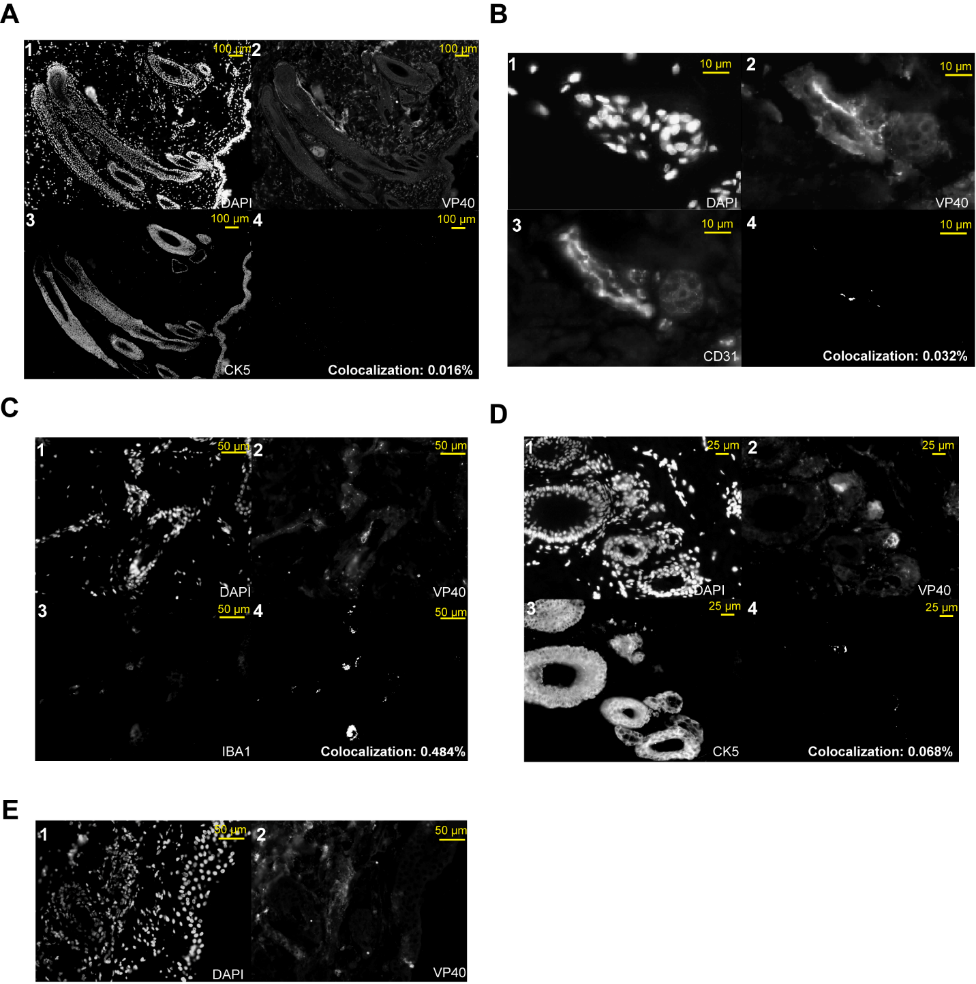


**Fig. S2. EBOV traffics to and infects NHP skin during systemic infection: single channel images and colocalization analyses for Figure 1.** **A–E**) Single-channel images of the skin section immunostaining from Fig. 1C–G, shown in grayscale. Colocalization was analyzed using the ImageJ Colocalization Highlighter plugin. Individual channels are shown in panels 1–3, and colocalization is shown in panel 4, with the percent area of colocalization displayed. Scale bar (μm) is shown in each panel.

**
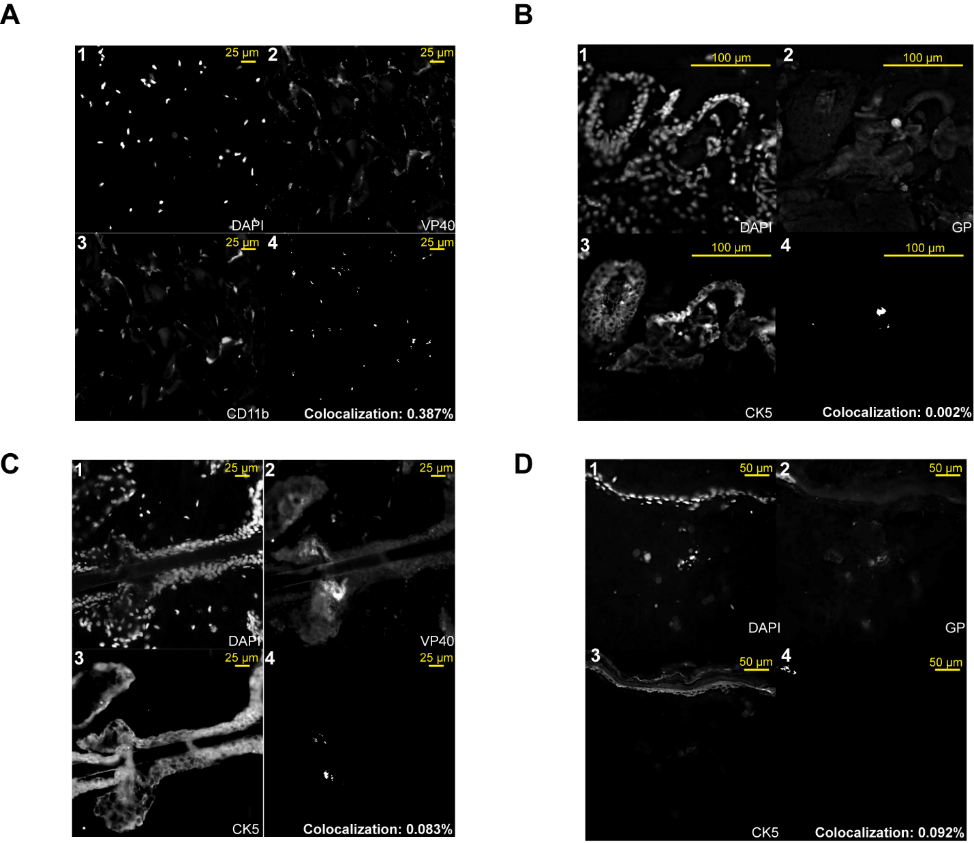
**

**Fig. S3. EBOV traffics to and infects NHP skin during systemic infection: single-channel images and colocalization analyses for Supplemental Figure 1.** **A–D**) Single-channel images of immunostained skin sections from Supplemental Fig. 1A–C and G, shown in grayscale. Colocalization was analyzed using the ImageJ Colocalization Highlighter plugin. Individual channels are shown in panels 1–3, and colocalization is shown in panel 4, with the percent area of colocalization indicated. Scale bar (μm) is shown in each panel.

**
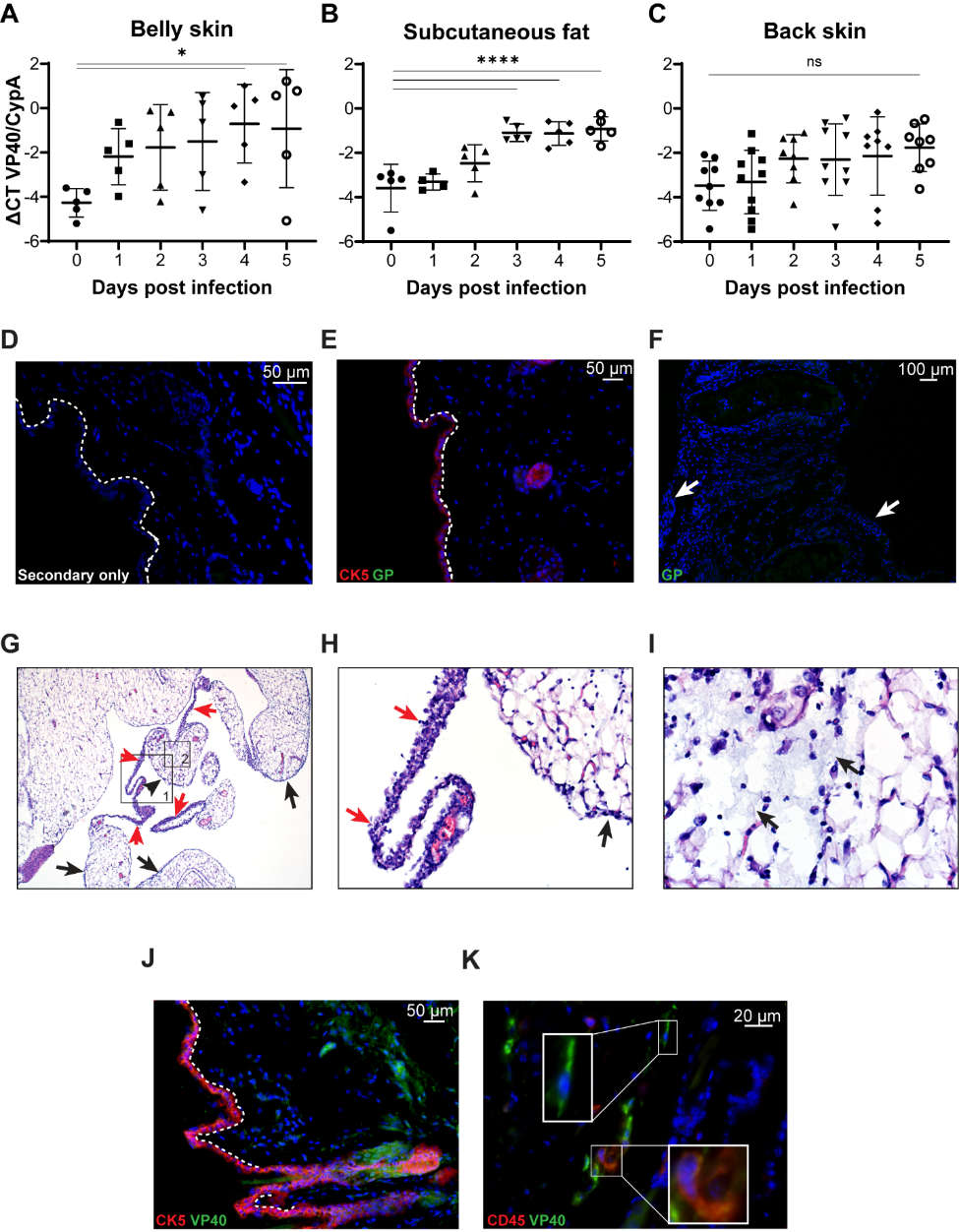
**

**Fig. S4. ma-EBOV kinetics and viral cell tropism in mice: staining controls and additional images.** C57BL/6 mice were infected, and tissues were collected on days 0-5 post infection as described in Fig. 2. **A–C**) Viral loads in belly skin (**A**), subcutaneous fat (**B**), and back skin (**C**) were measured by RT-qPCR for EBOV VP40 and normalized to the housekeeping gene, CypA. Data are expressed on a log_10_ scale as geometric means ± geometric SD. A one-way ANOVA was used to evaluate statistical significance (**A–C,** *p<0.05; ****p<0.0001). **D–F**) Representative immunostaining controls. **D**) Infected day 5 thigh tissue with secondary antibody alone. **E**) mock-infected thigh tissue stained for EBOV GP (green), CK5 (red), and DAPI (blue). **F**) Visceral fat from an uninfected mouse stained for EBOV GP shows no viral antigen. White arrows indicate suspected visceral ligaments. **G–I**) H&E staining of visceral ligaments in visceral fat from day 5 infected mice (corresponding to Fig. 2I). **G**) Inflammation and increased cellularity observed in visceral ligaments (red arrows) and the visceral peritoneal lining (black arrows). **H**) Inset 1 from G shows adipose-targeted inflammation (red arrows), with mononuclear cell and neutrophil infiltration and evidence of necrosis (black arrow). **I**) Inset two from G shows adipose-targeted inflammation and fat necrosis (black arrows). **J–K**) Immunostaining of thigh skin from ma-EBOV-infected mice. **J**) CK5 (red) and EBOV VP40 (green) shows viral antigen in sebaceous glands near hair follicles, without CK5 colocalization in day 5 infected thigh skin. **K**) CD45 (red) and EBOV VP40 (green) staining of day 4 thigh skin reveals elongated CD45^-^/VP40^+^ cells in the dermis (top inset), and focal clusters of VP40^+^ cells near a CD45^+^ cell (bottom inset). Brightness and contrast were adjusted uniformly across the entire image for visualization purposes. Scale bar (μm) is indicated in each panel. Single channels and colocalization analyses for panels **J–K** are shown in Supplemental Fig. 5.

**
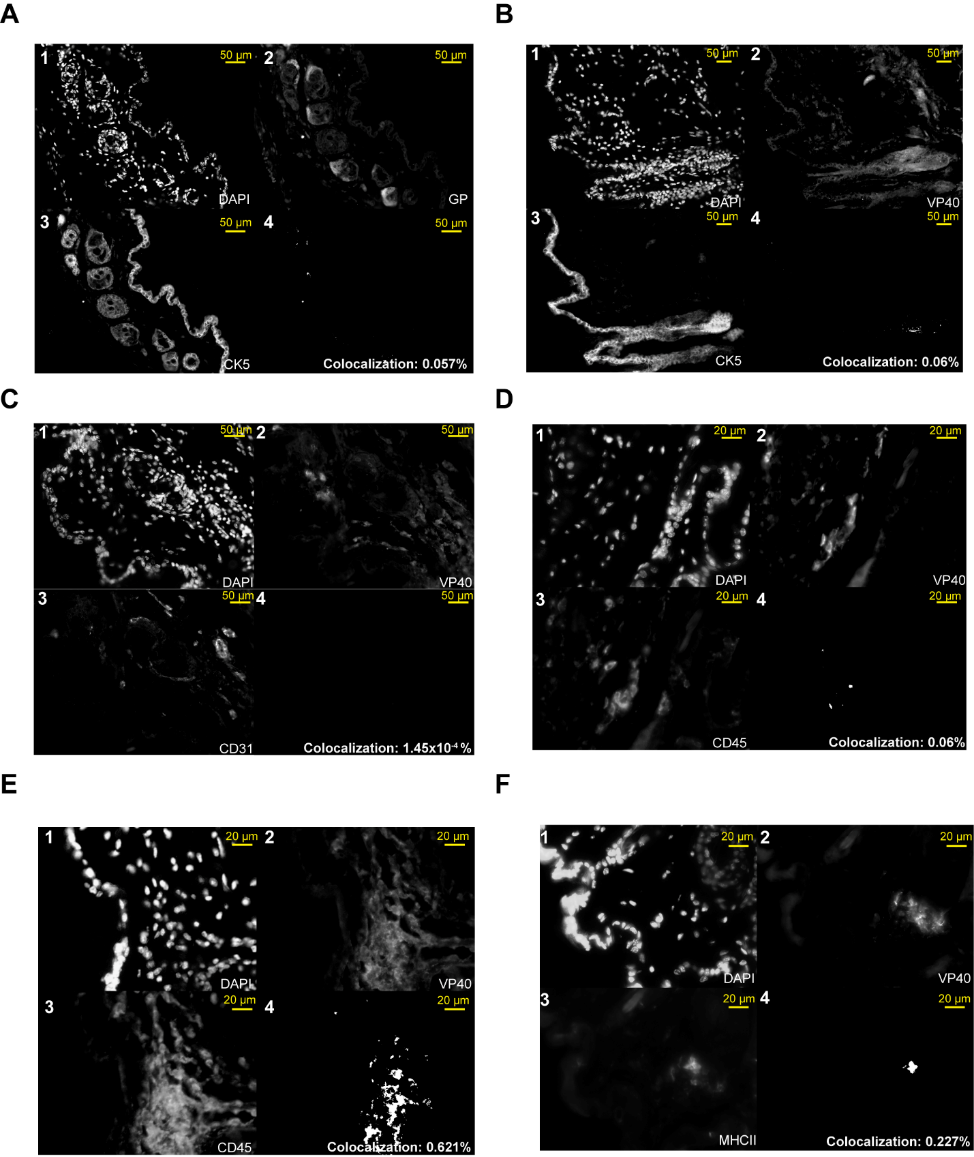
**

**Fig. S5. ma-EBOV kinetics and viral cell tropism in mice: single channel images and colocalization analyses for thigh skin panels in Figure 2 and Supplemental Figure 4.** **A–F**) Single-channel images of immunostained skin sections corresponding to Fig. 2J (**A**), Supplemental Fig. 4J (**B**), Fig. 2K (**C**), Supplemental Fig. 4K (**D**), Fig. 2L (**E**), and Fig. 2M (**F**), shown in grayscale. Colocalization was analyzed using the ImageJ Colocalization Highlighter plugin. Individual channels are displayed in panels 1–3, and colocalization is shown in panel 4, with the percentage area of colocalization indicated. Scale bar (μm) is shown in each panel.

**
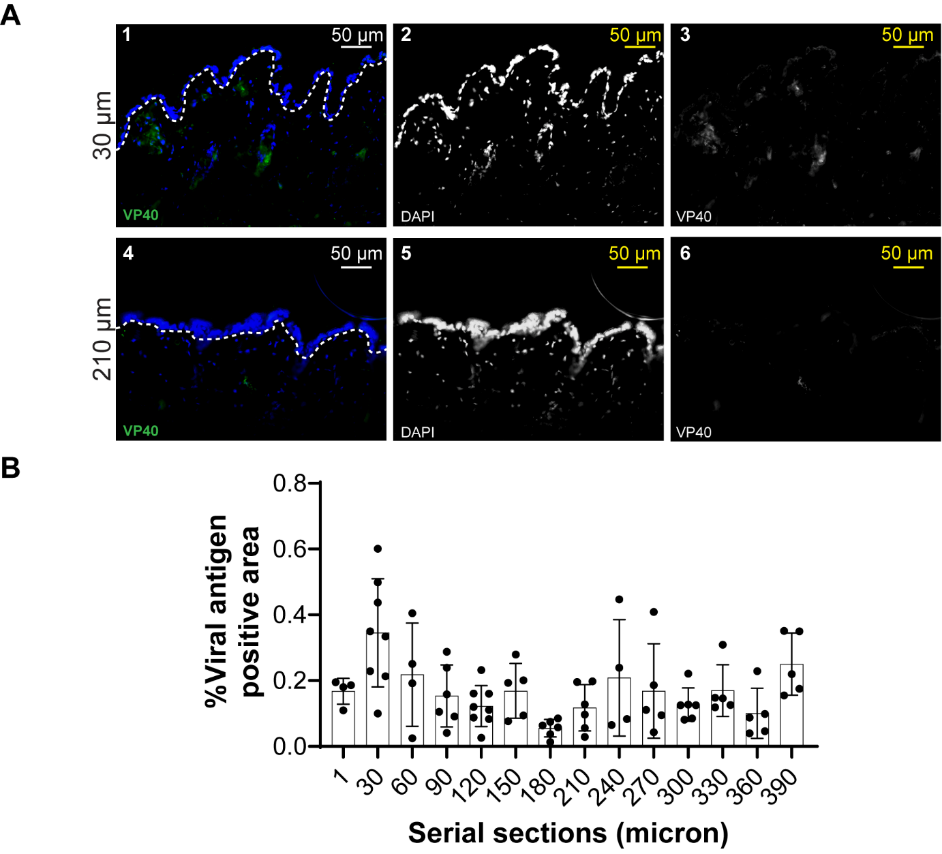
**

**Fig. S6. ma-EBOV kinetics and viral cell tropism in mice: serial sectioning of distal back skin from EBOV-infected *Ifnar ^–/–^*** **mice.** Distal back skin was collected from C57BL/6 *Ifnar ^–/–^* mice (n=5) infected IP with 1000 FFU of EBOV at the NEIDL and euthanized upon reaching endpoint criteria. **A–B**) Serial 30 μm sections were analyzed for EBOV staining across the tissue block. **A**) Representative sections from the highly positive 30 μm section and a minimally positive section located at 210 μm. Sections were immunostained for EBOV VP40 (green) and DAPI (blue). Composite images (panels 1 and 4) are shown on the left, with single channels in grayscale (panels 2–3 and 5–6) for DAPI (center) and VP40 (right). **B**) Percent area positive for VP40 was quantified in each section using ImageJ.

**
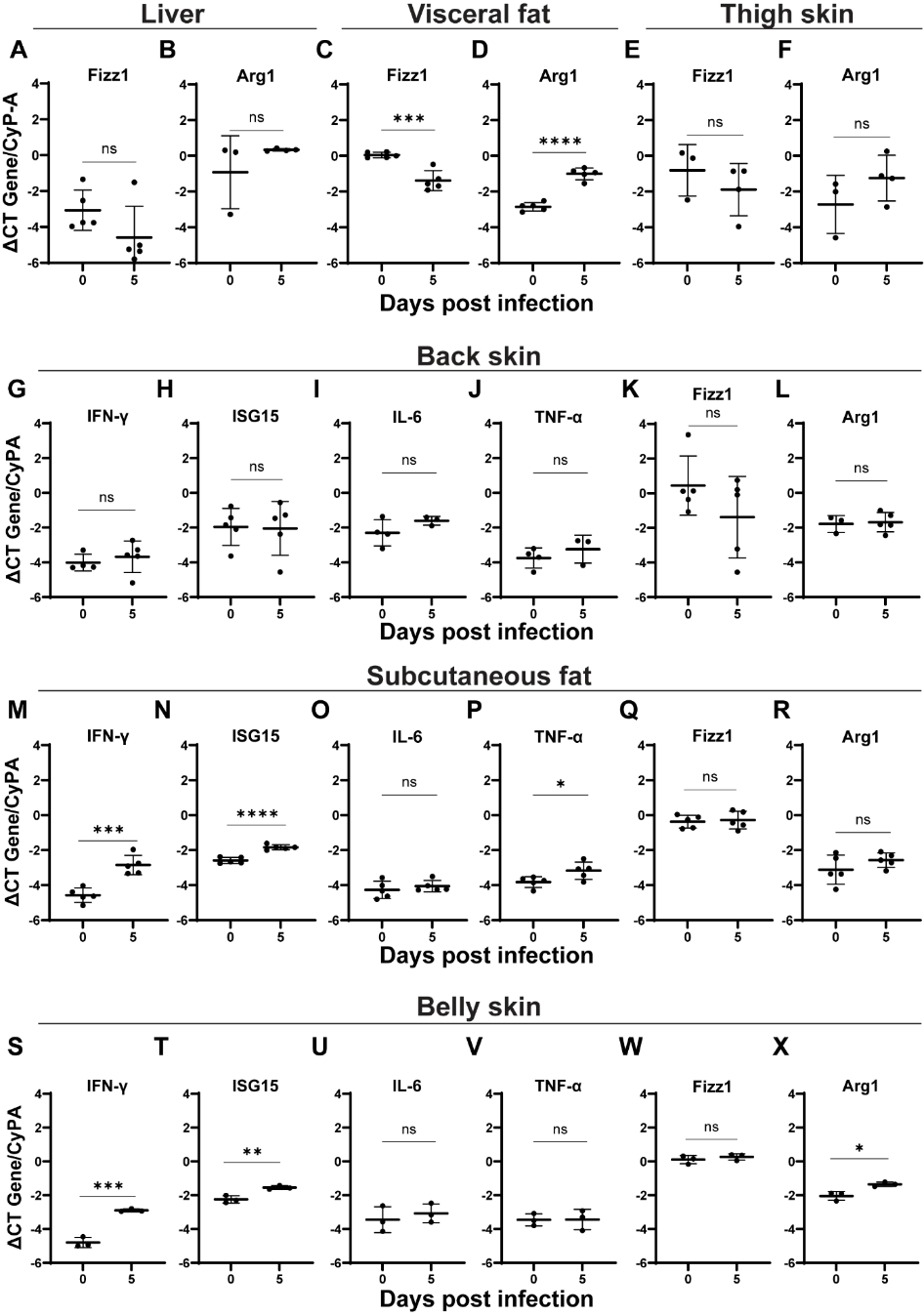
**

**Fig. S7. Inflammatory profiles of ma-EBOV-infected mouse tissues: additional tissue sites.** C57BL/6 mice were infected as described in Fig. 2. Expression of proinflammatory (IFN-γ, ISG15, IL-6, and TNF-α) and immunomodulatory (Fizz1 and Arg1) gene transcripts was evaluated by RT-qPCR and normalized to the housekeeping gene, CypA. Transcript levels were measured in the liver (**A–B**), visceral fat (**C–D**), thigh skin (**E–F**), back skin (**G–L**), subcutaneous fat (**M–R**), and belly skin (**S–X**) at 0 and 5 dpi. Data are shown on a log_10_ scale as geometric means ± geometric SD. Student’s t test determined statistical significance, (*p<0.05; **p<0.01; ***p<0.001; ****p<0.0001).

**
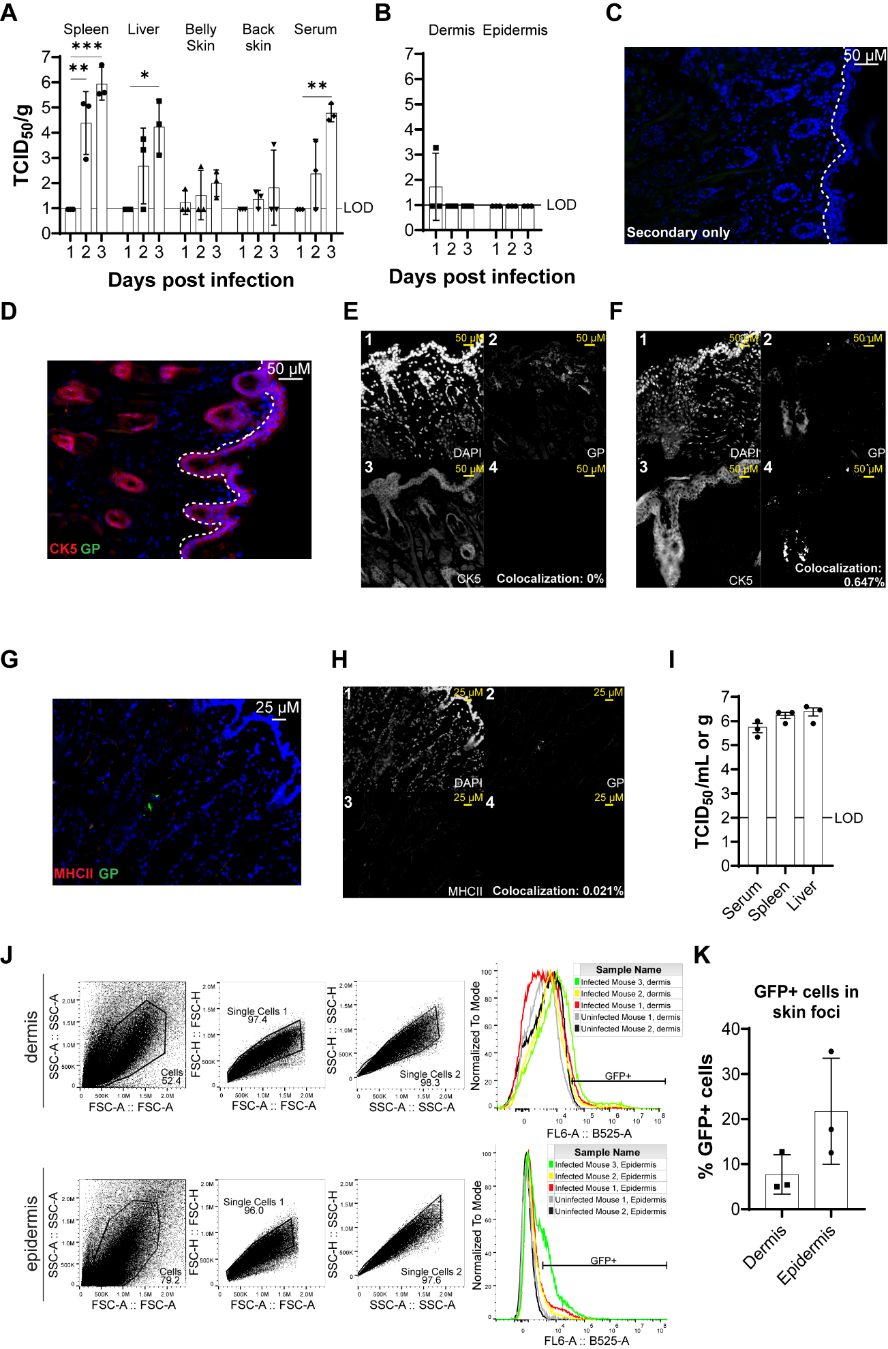
**

**Fig. S8. rVSV/EBOV GP targets similar cells as ma-EBOV in distal skin: rVSV/G titers, staining controls, and focal virus distribution in skin.** **A–B**) C57BL/6 *Ifnar ^–/–^* mice were infected IP with 1000 TCID_50_ of rVSV/G (n=3). Viral titers were measured from 1–3 days post infection in spleen, liver, belly skin, and back skin (**A**), and in enzymatically separated epidermal and dermal layers of back skin (**B**). Data are shown on a log_10_ scale as geometric means ± geometric SD. Statistical significance was determined for each tissue by one-way ANOVA on transformed values, (*p<0.05; **p<0.01; ***p<0.001). **C–D**) Representative images of infected day 3 cheek tissue stained with secondary antibody alone (**C**) and mock-infected thigh tissue stained with both primary and secondary antibody staining (**D**). The dotted line in panels **C** and **D** denotes the dermal-epidermal junction. **E–F)** Single channel images of immunostained skin from Fig. 4C–D shown in grayscale. Colocalization was analyzed using the ImageJ Colocalization Highlighter plugin. Individual channels are shown in panels 1–3 and colocalization in panel 4, with percent area of colocalization displayed. **G-H**) Back skin sections were stained for EBOV GP (green), MHC II (red), and DAPI (blue) (**G**), with corresponding single-channel images in white (panels 1-3) and colocalization shown in panel 4, with percent area of colocalization displayed (**H**). Brightness and contrast were adjusted uniformly across all images for visualization purposes. Scale bar is indicated in each panel (μm). **I**) Viral titers in serum, spleen, and liver confirm systemic infection in mice infected IP with rVSV/EBOV GP (shown in Fig. 4F). **J–K**) 6–8-week-old male C57BL/6 *Ifnar ^–/–^* mice were infected IP with 500 TCID_50_ of rVSV/EBOV GP (n=3) and euthanized 3 days post infection. GFP-bright foci were visualized by fluorescence and isolated via 3 mm biopsy punches. Flow cytometry was performed on epidermal and dermal single cell suspensions to assess GFP positivity. Gating strategy and representative histograms are shown (**J**), with summary data displayed (**K**).

**
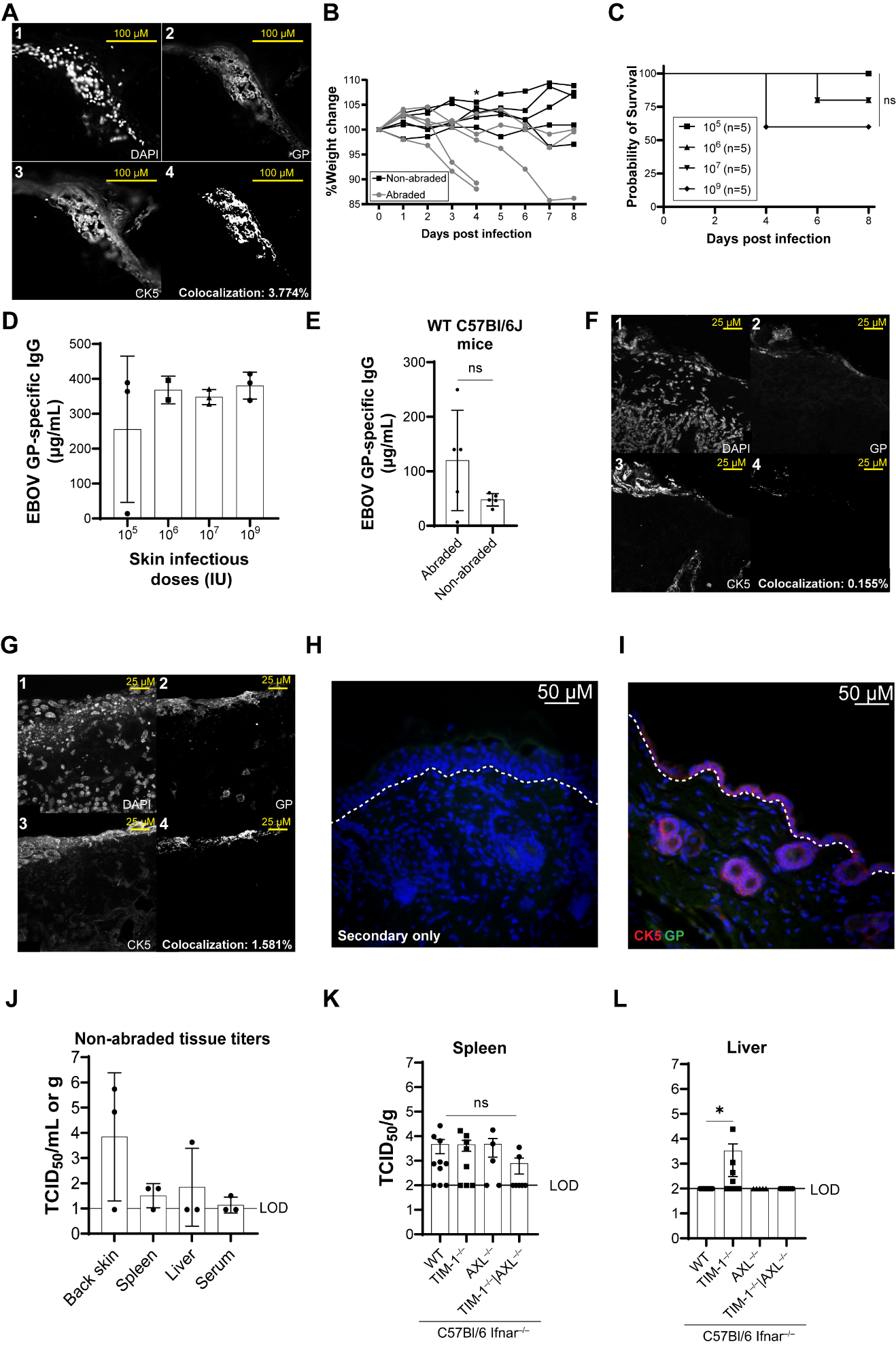
**

**Fig. S9. Infection of the epidermal surface of skin results in viral dissemination and morbidity: survival curve, IgG production and staining controls. A**) Single-channel images from immunostained skin in Fig. 5B are shown in grayscale. Colocalization analysis was performed using the ImageJ Colocalization Highlighter plugin. Individual channels are shown in panels 1–3 and colocalization in panel 4, with the percent area of colocalization displayed. **B**) Weight loss measured in C57BL/6 *Ifnar ^–/–^* mice infected as described in Fig. 5C. Data are expressed as percent weight loss from baseline. Statistical significance was determined per day by student’s T test (*p<0.05). **C–D**) C57BL6/J *Ifnar ^–/–^* males (n=5) were infected topically on abraded back skin with rVSV/EBOV GP (10^5^ - 10^9^ TCID_50_ in 10 μL DPBS) of rVSV/EBOV GP. Survival over 8 days (C) and serum anti-EBOV GP IgG titers at 21 dpi (D) were measured. Significance was determined by log-rank test. **E**) WT C57BL6/J male mice (n=5) were treated topically with rVSV/EBOV GP (10^9^ TCID_50_ in 10 µL DPBS) on either abraded or non-abraded back skin and bled 21 days post inoculation to assess anti-EBOV GP IgG antibody titers. **F**) C57BL6/J *Ifnar ^–/–^* (n=3) mice were treated with rVSV/EBOV GP (10^9^ TCID_50_ in 10 µL DPBS) on non-abraded. Viral titers were measured in back skin, spleen, liver, and serum at 24 hours post infection. **G–H**) Single channel images from Fig. 5F (**G**), and Fig. 5G (**H**) are shown in grayscale (panels 1–3), with colocalization in panel 4 and percent area of colocalization displayed. **I–J**) Representative staining controls from 24-hour post-infection back tissue with secondary antibody alone (**I**) and mock-infected back skin (**J**). Brightness and contrast were adjusted uniformly across the entire image for visualization purposes. Scale bar (μm) is shown in each panel. **K–L**) Viral titers quantified in spleen (**K**) and liver (**L**) from the same mice described in Fig. 5K-L. Statistics were determined by one-way ANOVA on transformed values (*p<0.05).
